# Designing Stable Graphitic Networks on Ultra‐Porous Polyimide Aerogels via Solvent‐Guided Structuring

**DOI:** 10.1002/smll.202505776

**Published:** 2025-12-12

**Authors:** Tingting Wu, Mengmeng Li, Mingxiang Gao, Christopher H. Dreimol, Ekaterina Filimonova, Qin Li, Yunhong Wang, Dimitrios Sapalidis, Michal Ganobjak, Yanfang Wei, Joshua Yip, Bruno F. B. Silva, Anja K. Skrivervik, Wim J. Malfait, Shanyu Zhao

**Affiliations:** ^1^ Laboratory for Building Energy Materials and Components Empa, Swiss Federal Laboratories for Materials Science and Technology Überlandstrasse 129 Dübendorf 8600 Switzerland; ^2^ Institute of Sustainability for Chemicals Energy and Environment (ISCE2) Agency for Science, Technology and Research (A*STAR) 1 Pesek Road Jurong Island 627833 Singapore; ^3^ Microwaves and Antennas Group Institute of Electrical and Micro Engineering École Polytechnique Fédérale de Lausanne Lausanne 1015 Switzerland; ^4^ Wood Materials Science Institute for Building Materials ETH Zürich Zurich 8093 Switzerland; ^5^ Cellulose & Wood Materials Laboratory Empa, Swiss Federal Laboratories for Materials Science and Technology Überlandstrasse 129 Dübendorf 8600 Switzerland; ^6^ IBIH Advanced Material Co., Ltd Lingang Economic And Technological Development Zone Cangzhou 061108 China; ^7^ Center for X‐ray Analytics Empa, Swiss Federal Laboratories for Materials Science and Technology Lerchenfeldstrasse 5 St. Gallen 9014 Switzerland; ^8^ Laboratory for Biointerfaces Swiss Federal Laboratories for Materials Science and Technology Lerchenfeldstrasse 5 St. Gallen 9014 Switzerland; ^9^ Laboratory for Biomimetic Membranes and Textiles Empa, Swiss Federal Laboratories for Materials Science and Technology Lerchenfeldstrasse 5 St. Gallen 9014 Switzerland

**Keywords:** aerogels, laser‐induced graphene, patch antenna, polyimide, thermal management

## Abstract

Lightweight, highly porous polyimide (PI) aerogels have emerged as promising candidates for advanced electronic applications due to their exceptional thermal stability, mechanical performance, structural integrity, and low dielectric loss. However, the controlled laser‐induced graphitization (LIG) of such ultra‐porous polymeric networks remains a critical challenge, as localized high temperatures often trigger polymer backbone degradation and framework collapse. Herein, a chemically engineered PI aerogel via a molecular design strategy that tailors solvent–polymer interactions during gelation to produce a hierarchically porous yet thermally robust network is reported. This substrate preserves its porosity and integrity during high‐intensity LIG, enabling the formation of a uniform graphene–carbon conductive phase embedded within the polyimide matrix. The resulting material achieves sheet resistivity as low as 6.5 Ωsq^−1^, while retaining excellent dielectric properties (ɛ_r_ = 1–2, tan δ <0.2) and thermal insulation (30–35 mW m^−^^1^ K^−^^1^ post‐300 °C treatment). This synergy between molecular design, thermal response, and electronic functionality enables integration into multifunctional devices, such as flexible pressure sensors, thermal management layers, and ultralight antennas, demonstrated by a reflection coefficient of −14 dB at 5.4 GHz and a peak gain of 3.9 dBi.

## Graphical abstract

A thermally robust, hierarchically porous polyimide aerogel is engineered via solvent‐polymer interaction tuning to enable stable laser‐induced graphitization. The resulting graphene‐integrated structure exhibits low resistivity, excellent dielectric performance, and thermal insulation, supporting applications in flexible electronics, thermal management, and lightweight antennas.

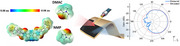

## 1 Introduction

Materials with extreme properties, such as high conductivity for thermal and electrical energy, e.g. metals or advanced 2D materials like graphene,^[^ ^1^, ^2^, ^3^ ^]^ or exhibiting super insulation characteristics, e.g. ceramics or polymers,^[^ ^4^, ^5^ ^]^ play crucial roles in flexible electronics, energy storage, and aerospace components. Combining highly conductive and super‐insulating properties into a single product holds significant potential benefits across various industries. In high‐power electronic devices or circuits where efficient heat dissipation and electrical conductivity are paramount, such a combination can offer dual functionality^[^ ^6^ ^]^ and enhance performance and reliability through improved energy efficiency, thermal management, and more compact designs. However, combining conductive and non‐conductive materials poses notable challenges: it is far from trivial to achieve material compatibility and ensure proper bonding and structural integrity.^[^ ^7^ ^]^ Additionally, maintaining the desired balance between conductivity and insulation properties can be challenging, as changes to one property may inadvertently affect the other. Moreover, manufacturing processes for such composite materials are complex and costly, necessitating precise control over material composition and processing parameters. Despite these challenges, specific applications can greatly benefit from materials with combined properties. For instance, patch antennas, essential components in wireless communication systems, require conductive and insulating properties.^[^ ^8^ ^]^ Incorporating a material with high electrical conductivity and low dielectric constant into the patch antenna design allows optimal signal transmission and reception with minimal energy loss and interference.

In terms of extremely low thermal conductivity and dielectric constant, polyimide aerogel emerges as one of the most promising materials for various applications,^[^ ^9^, ^10^, ^11^ ^]^ including thermal insulation in batteries,^[^ ^12^ ^]^ lightweight dielectric insulators in power cables,^[^ ^13^ ^]^ and as a dielectric substrate for antenna.^[^ ^7^ ^]^ Previously, lightweight patch antenna utilizing polyimide aerogel substrates with copper patterns has demonstrated broader bandwidth and higher gain while maintaining significantly lower mass compared to commercial antenna substrates, such as polytetrafluoroethylene (PTFE), ceramics, and epoxy‐based materials.^[^ ^7^ ^]^ However, it is important to address issues such as delamination and degradation in the conductive copper pattern layer, as these negatively impact the performance and reliability of the patch antenna, resulting in signal loss, decreased efficiency, and potential antenna failure.

A promising approach for adding a conductive layer to commercially available dense polyimide films is laser‐induced graphitization (LIG).^[^ ^14^, ^15^, ^16^, ^17^ ^]^ This technique involves thermochemical reactions induced by a high‐energy laser, which reorganizes C─O, C═O, N─C, and aromatic rings in polyimide.^[^ ^18^, ^19^ ^]^ However, this technology cannot be directly applied to conventional mesoporous polyimide aerogels despite their ideal properties, such as ultra‐low density (0.05–0.15 g cm^−3^) and extremely low dielectric constant.^[^ ^20^ ^]^ This limitation is primarily due to the deterioration of the physical structure (pore collapse) when exposed to temperatures above 150 °C,^[^ ^21^ ^]^ well below the glass transition or decomposition temperature.^[^ ^22^, ^23^ ^]^ Laser scribing, a high‐power sintering process, requires polyimide aerogel substrates with excellent chemical and dimensional stability to ensure the formation of a continuous graphene‐like carbon layer. This stability cannot be achieved by the typical method of incorporating high‐temperature‐resistant components, such as cellulose nanocrystals,^[^ ^24^ ^]^ attapulgite,^[^ ^25^ ^]^ or silica aerogels,^[^ ^21^, ^26^ ^]^ which can improve dimensional stability under high temperature but hinder the formation of continuous graphene layers during LIG, resulting in insufficient conductivity. High electrical conductivity is essential for the effective performance of patch antennas. Therefore, designing a polyimide aerogel with a robust porous structure that withstands high‐power laser scanning while enabling the formation of high‐quality graphene layers is essential for its application in patch antennas.

## 2 Results and Discussion

### 2.1 Solvent‐Guided Structuring of Heat‐Resistant Polyimide Aerogel Substrates for Conductive Graphitic Networks

**Figure 1:**
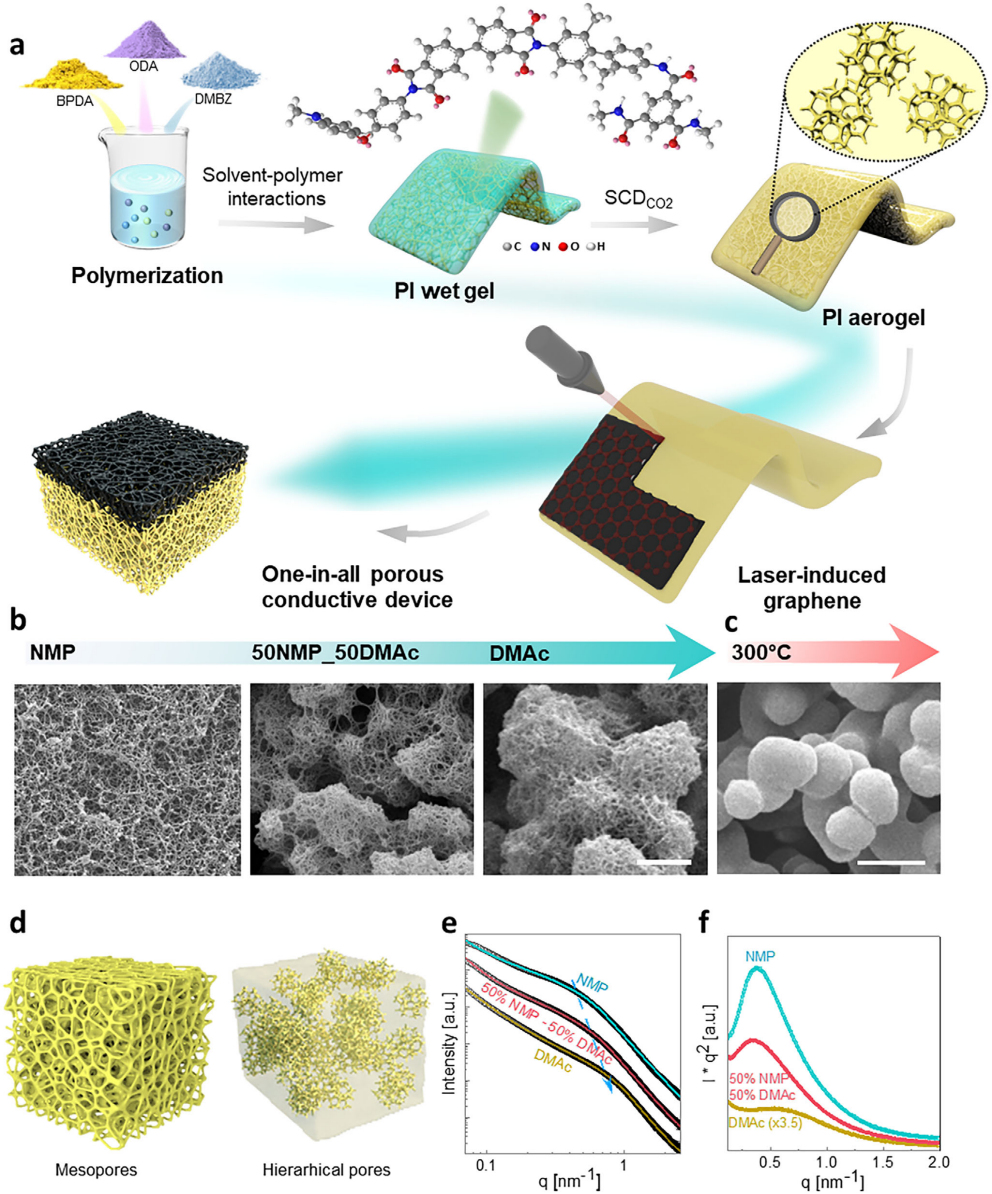
Synthesis of hierarchical polyimide aerogels. a) Synthesis of polyimide aerogels with backbones of BPDA‐DMBZ‐ODA. b) Scanning electron microscopy (SEM) images of mesoporous (PI_NMP_, 0 vol.% DMAc), hierarchical (50 vol.% DMAc), and spherical polyimide aerogel (PI_DMAc_, 100 vol.% DMAc) dried through SCD (scale bar 2 µm). c) SEM of PI_DMAc_ after 24 h at 300 °C (scale bar 2 µm). f) Scheme of phase separation processes. e) SAXS curve. (f) Kratky profile

In this study, we proposed a molecular design approach that adjusts the rigidity m‐tolidine (DMBZ) or flexibility 4, 4'‐oxydianiline (ODA) of segments within the polyimide chains (**Figure** **1**a). Additionally, we focused on optimizing the packing arrangement between polymer chains by modulating the solubility of polyimide in different solvents and their mixtures (x% N‐methylpyrrolidone (NMP) + y% dimethylacetamide (DMAc), with x:y volume ratios of 10:0, 5:5, 3:7, 1:9, and 0:10), while keeping other parameters. In different solvents, the microstructures of the polyimide aerogels evolve from nanometer‐scale, mesoporous structures comprised of polymer nanofibers to micrometer‐scale structures comprised of macropores and porous aggregates of accumulated nanofibers (Figure 1b; Figures S1 and S2, Supporting Information). This variation in microstructures originates from the various polymer‐solvent interactions in different solvents as the poly (amic acid) converts into polyimide during the imidization. Interestingly, the micrometer‐scale structures impart enhanced stability, particularly at high temperatures (Figure 1c), microscopically, only the NMP sample exhibits a homogeneous microporous structure. With the addition of DMAc, all samples display hierarchical porosity, including larger pores in the micrometer range (Figure S3, Supporting Information). In preliminary tests with other monomer systems, such as those based on ODA alone, the resulting aerogels consistently exhibited mesoporous structures regardless of solvent ratio. Only the BPDA‐ODA/DMBZ (5:5) formulation demonstrated a unique and systematic sensitivity to the DMAc/NMP ratio, particularly within the 1:9–9:1 range.

To delve deeper into the gel formation mechanism, we conducted small‐angle X‐ray scattering (SAXS) analysis on the polyimide aerogels formed in pure NMP, a 50–50 mixture, and pure DMAc (Tables S1 and S2, Supporting Information), with the corresponding microstructures described in Figure 1d. Two *q* regions can be distinguished. Independent of the solvent system, the high‐*q* region displays the ideal Porod slope of d*log(I)/*d*log(q) ≈*−4, related to the presence of a two‐phase system with sharp interfaces where the scattering length density can be described by step functions.^[^ ^27^ ^]^ The low‐*q* region (with a smaller slope) extends to higher *q* values as the DMAc content increases (Figures S4 and S5, Supporting Information). The slope d*log(I)/*d*log(q)* in the low‐*q* region varies from *≈*−2 to −1.4. The observed upturns in the low‐*q* region, particularly noticeable in the DMAc sample, are due to mass fractals formed by locally assembled nanofibers during the faster gel formation (Figure S6 and Movie S1, Supporting Information), which induce densified regions that significantly contribute to the scattering intensity.^[^ ^28^ ^]^ The configuration is also evident in the Kratky plot (Figure 1e). The NMP sample's Kratky plot exhibits a peak maximum followed by a decrease and a plateau at high q, which is an indication of some inhomogeneities or branched chains.^[^ ^29^ ^]^ For the DMAc sample, after the presence of a peak, there is an upturn in the low q region, which, as aforementioned, originates from the strong contribution of the mass fractal due to more local densification.

**Figure 2:**
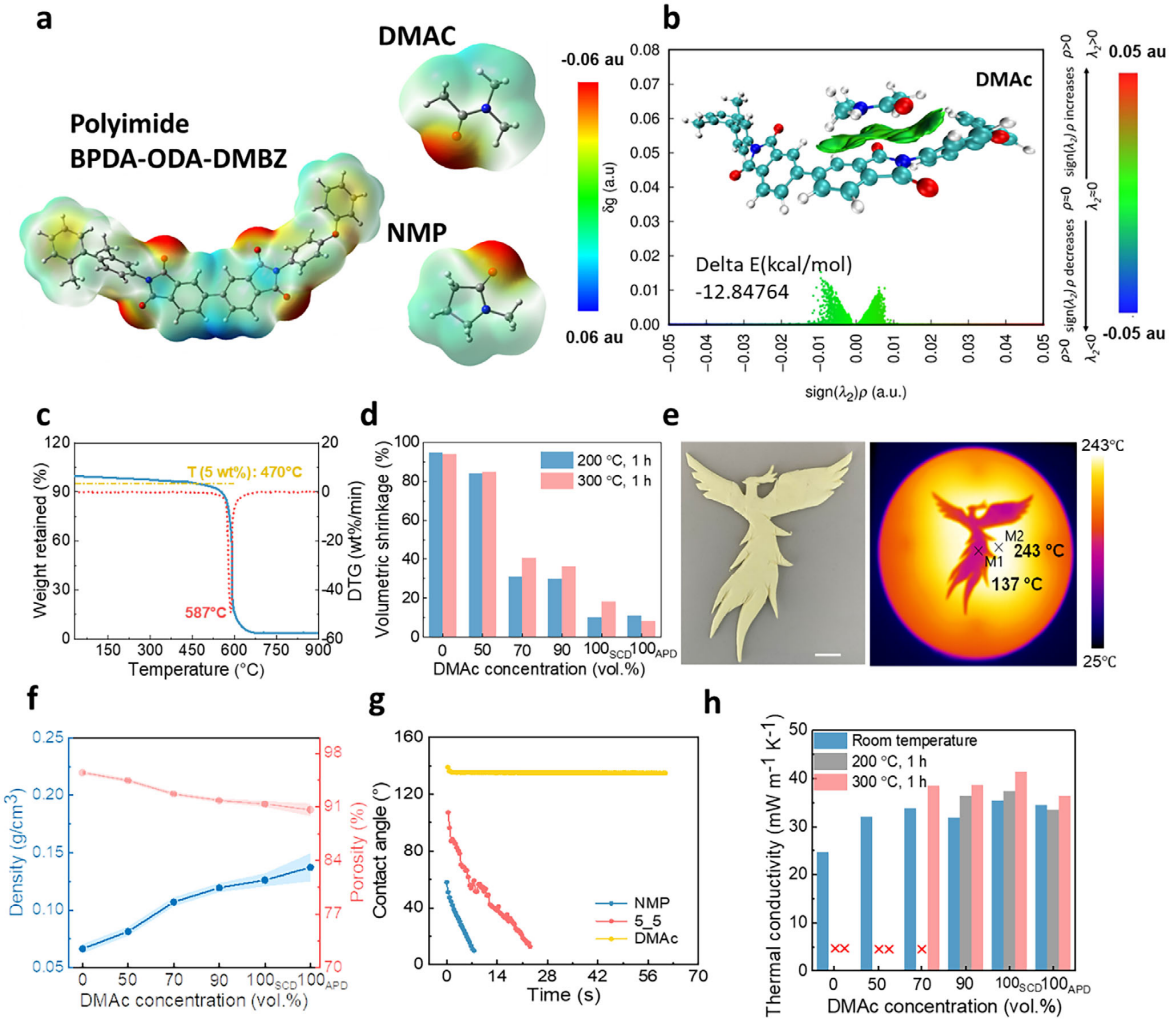
Tailorable microstructure formation mechanism and their properties. a) Electrostatic potential distributions of different solvents and polyimides. b) Schematic diagram and the IGMH results of the interaction between defined polyimide structure and DMAc. c) TGA of polyimide aerogels (PI_NMP_) in air. d) Volumetric shrinkage after thermal treatment at 200 and 300 °C for 1 h of polyimide aerogels prepared in different solvents. e) Photo of a phoenix cut from a large PI_DMAc_ aerogel sheet (2.2 mm thickness) and infrared (IR) image on a thermal stage (scale bar 10 mm). f) Density and pore volume of polyimide aerogels made from different solvents. g) Water contact angle evolution with time. h) Thermal conductivity of polyimide aerogels before and after thermal treatment.

To understand the different microstructure formations, the mutual interaction forces of the single block (BPDA‐DMBZ‐ODA) oligomer in the two solvents were simulated using density functional theory (DFT) (**Figure** **2**a).^[^ ^30^ ^]^ The highly conjugated polyimide, with its flexible molecular chains and large free volume, exhibits good compatibility with both NMP and DMAc solvents. The Independent Gradient Model based on Hirshfeld partition (IGMH) analysis indicates that the interactions between BPDA‐DMBZ‐ODA molecules and both solvents are primarily governed by van der Waals forces (Figure 2b; Figure S7, Supporting Information). The binding energy (*∆E*) with NMP is −14.01 kcal mol^−1^, slightly lower than with DMAc at −12.86 kcal mol^−1^, suggesting a marginally stronger binding force between the polymer and NMP, thereby promoting greater stability. This slight difference in *∆E* facilitates slow and homogeneous gel formation in NMP, whereas faster gelation in DMAc leads to the formation of a coarser structure.

### 2.2 Properties of Polyimide Aerogels with Tailorable Microstructures

Despite the dramatic differences in morphology, polyimide aerogels exhibit excellent thermal stability in their chemical structure (T_5wt.%_ of 466 °C, Figure 2c), largely due to the presence of imide rings and aromatic structures in the backbone.^[^ ^21^ ^]^ However, the homogeneous, nanoscale aerogel (PI_NMP_) displayed poor dimensional stability due to the collapse of its mesoporous structure upon heating (Figure 2d). By increasing the DMAc content and modifying the structure from mesoporous to hierarchical and then to spherical, dimensional stability was significantly improved, with reduced shrinkage (Figure 2d). Specifically, mesoporous aerogels (PI_NMP_) experienced over 90% volumetric shrinkage after 1 h of exposure at 200 and 300 °C, while spherical porous aerogels (PI_DMAc_) showed only 10% and 18% shrinkage, respectively. SEM investigation of the PI_DMAc_ aerogel after 24 h at 300 °C revealed that the mesoporous nanofiber networks within the spheres (Figure 1b) fused into non‐porous spheres with smooth surfaces (Figure 1c), but the network of micrometer‐sized spheres remained intact, imparting the observed low volumetric shrinkage.

Moreover, the hierarchical and spherical aerogels (formed with more DMAc) were softer (Figures S8 and S9, Supporting Information) and exhibited superior flexibility compared to the mesoporous aerogels (formed with more NMP) (Figure S10 and Movie S2, Supporting Information), along with excellent machinability and a high potential for subtractive manufacturing (Figure 2e). Although the overall mechanical properties were significantly enhanced, the tearing and stretching resistance remain limited, likely due to the inherently high porosity of the materials. Nitrogen sorption data (Figures S11 and S12, Supporting Information) support the SEM observations (Figure 1b), with a strong decrease in BET surface area with increasing DMAc content, from 430 m^2^ g^−1^ for the mesoporous PI_NMP_ to 40 m^2^ g^−1^ for the micro‐spherical PI_DMAc_ and similar trends are observed for the BJH pore volume. With increasing DMAc concentration, the aerogel density increased from 0.06 g cm^−3^ to 0.13 g cm^−3^, with a corresponding decrease in porosity from 94% to 90% (Figure 2f). This shows that despite the use of identical polymer concentrations in the various preparations, the use of different solvents induces variable shrinkage and densification during phase separation and gelation.

One surprising finding is the significant improvement in surface hydrophobicity in the spherical PI_DMAc_ structure (with a stable water contact angle of 135°) compared to the mesoporous PI_NMP_, despite their identical polymer chemistry (Figure 2g). This strong water repellency likely results from Cassie‐Baxter states associated with the hierarchical, micro‐spherical structure (Figure 1b) which increases surface roughness at multiple length scales (Figure S13, Supporting Information).^[^ ^31^ ^]^ The mesoporous PI_NMP_ aerogel exhibits a lower room temperature thermal conductivity (25 mW m^−1^ K^−1^) compared to the more macroporous aerogels produced with DMAc or NMP‐DMAc mixtures (30–35 mW m^−1^ K^−1^) (Figure 2h), which is expected due to the Knudsen effect in the PI_NMP_’s mesopores. However, PI_NMP_ loses its low thermal conductivity after exposure to high temperatures due to excessive shrinkage (Figure 2d). In contrast, the spherical PI_DMAc_ structure shows the least degradation in insulation performance, with a room‐temperature thermal conductivity of 40 mW m^−1^ K^−1^ after 1 h at 300 °C. The shape stability and insulating performance was also demonstrated on a hot plate and using IR imaging (Figure 2e). Furthermore, PI_DMAc_, prepared via ambient pressure rather than supercritical drying, maintained a thermal conductivity below 35 mW m^−1^ K^−1^ after exposure to 300 °C (Figure 2h). These thermal conductivity results align with the top surface temperatures of aerogels placed on a 320 °C hotplate (Figure S14, Supporting Information).

In summary, by controlling the solvent during the gelation process (Figure 2b; Figure S7, Supporting Information), it is possible to synthesize polyimide aerogels with hierarchical, micro‐spherical structures that offer much greater dimensional stability at high temperatures, significantly increased hydrophobicity, and vastly improved flexibility and machinability.

### 2.3 Laser‐Induced Graphitization for Stable Conductive Paths on a Porous Matrix

The LIG laser, a high‐power heating process, rapidly converts dense highly stable polymers into graphene‐like carbon through thermochemical reactions.^[^ ^32^, ^33^ ^]^ Due to the highly insulating nature of the micro‐spherical PI_DMAc_ aerogel and its exceptionally low thermal conductivity (≈30 mW m^−1^ K), the surface temperature can rise sharply in a very short time when exposed to laser fluence in the range of 6.5 to 8.5 J cm^−^^2^. This rapid and intense heating significantly enhances the degree of graphitization on the surface. Moreover, the intrinsic thermal stability of the PI_DMAc_ aerogel ensures that the elevated temperatures generated during laser treatment do not collapse or damage the delicate porous structure – an essential factor for preserving both mechanical performance, structural integrity and large pore area.

**Figure 3:**
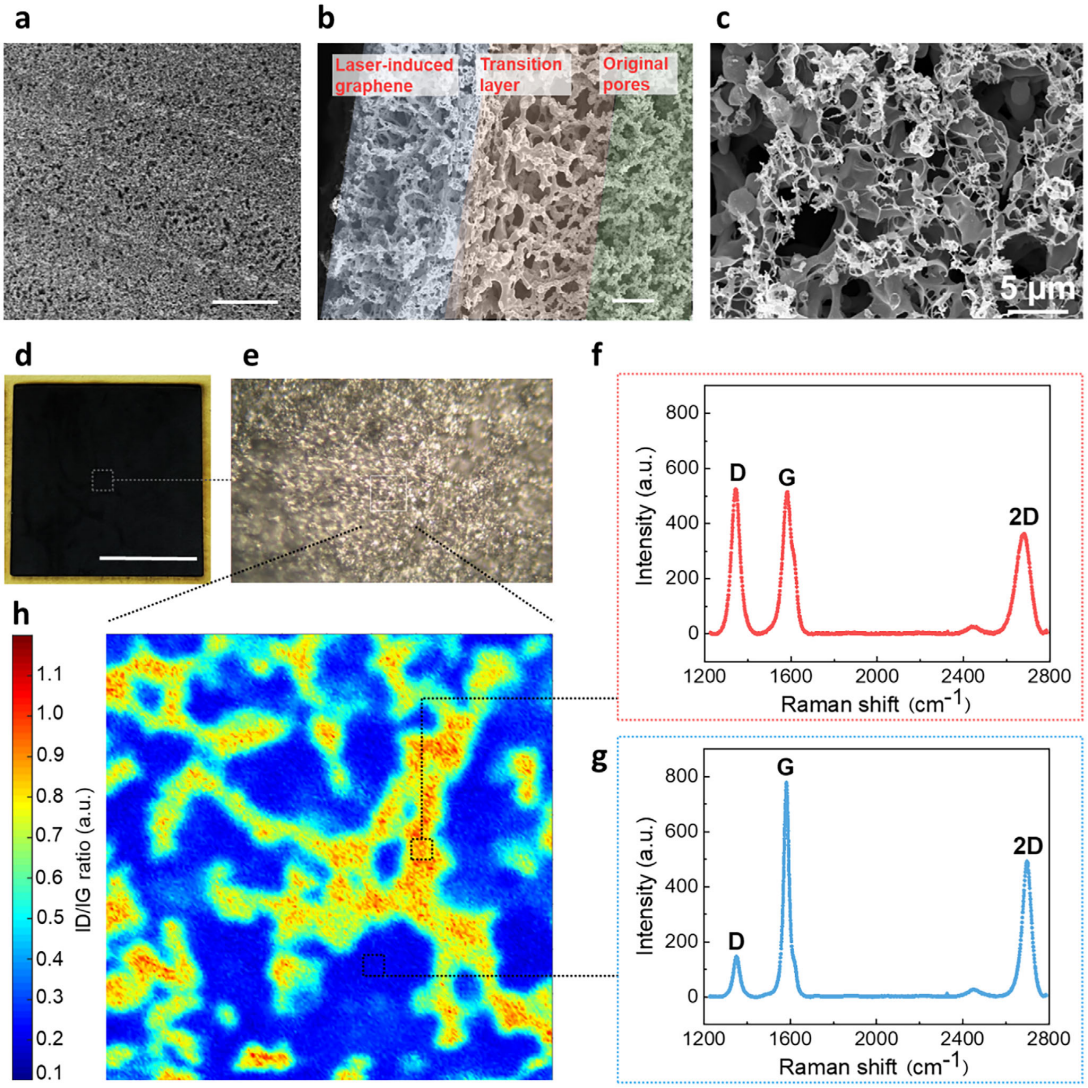
Laser‐induced graphene on polyimide aerogels (PI_DMAc_). SEM images of a) polyimide aerogel surface with LIG scribing marks (scale bar 100 µm), b) the cross‐section of the LIG layer and polyimide aerogel substrate (scale bar 20 µm), c) the surface of porous graphene‐like carbons (scale bar 5 µm). d) Photo of LIG layer on polyimide substrate (20 × 20 mm^2^, scale bar 10 mm). e) Optical microscope image of a selected area of the LIG layer (scale bar 50 µm). f,g) Raman spectra of two selected spots representing two extremes in terms of D (≈1350 cm^−1^) and G (≈1580 cm^−1^) peak intensity. h) Raman map of the D/G intensity ratio (size of the selected mapping range is 50 × 50 µm^2^).

Following laser scanning, the aerogel surface retains a microporous architecture without any noticeable thermal deformation (**Figure** **3**a). A cross‐section image (Figure 3b) reveals three distinct structural zones: on the right, the unaltered spherical structure of PI_DMAc_; in the middle, a transition region where the spheres have partially melted and merged into larger interconnected skeletons with expanded pores; and on the left, the laser‐exposed surface characterized by smaller, compact skeleton structures. A magnified surface image confirms the formation of a uniform, interconnected porous graphene layer (Figure 3c). Optical and SEM images further demonstrate that the entire surface of the polyimide aerogel undergoes consistent and homogeneous graphitization during laser treatment (Figure 3d,e).

Raman mapping and associated peak analysis (Figure 3f–h) provide insights into the graphitization degree of the polyimide aerogel substrate. Typically, graphene‐like carbons exhibit three characteristic peaks: the D‐peak at ≈1350 cm^−1^, the G‐peak at ≈1580 cm^−1^, and the 2D peak at ≈2970 cm^−1^. The G‐band corresponds to the vibration of sp^2^‐hybridized carbon, while a relatively weak D‐band indicates high‐quality graphene with fewer defects. The intensity ratio between the D and G peaks (I_D_/I_G_) is used to map the graphitization degree and assess the efficiency of the LIG process. Two representative spots from the Raman map (Figure 3h) were selected. The yellow region (Figure 3f) shows a higher D‐band intensity and, hence, a higher density of defects on the graphene layer, in contrast to the blue region (Figure 3g). The I_D_/I_G_ intensity map reveals a well‐connected network with a high degree of graphitization across the entire laser‐treated region, consistent with the surface SEM image (Figure 3c). This network provides an effective percolation pathway, resulting in low resistivity, as reflected by the low sheet resistivity of the graphene‐like carbon layer of ≈6.5 Ωsq^−1^.

### 2.4 Thermal and Electrical Conductivity Management

The micro‐spherical polyimide aerogel substrate is an excellent electrical and thermal insulator (Table S3, Supporting Information) and amenable to LIG, thanks to its exceptional dimensional stability. By customizing the formation of highly conductive graphene layers on these insulating substrates, we developed a versatile materials technology with transformative potential across multiple high‐value industries.^[^ ^34^ ^]^ Below, we demonstrate innovative application examples that utilize well‐defined conductive patterns on a highly insulating porous substrate.

**Figure 4:**
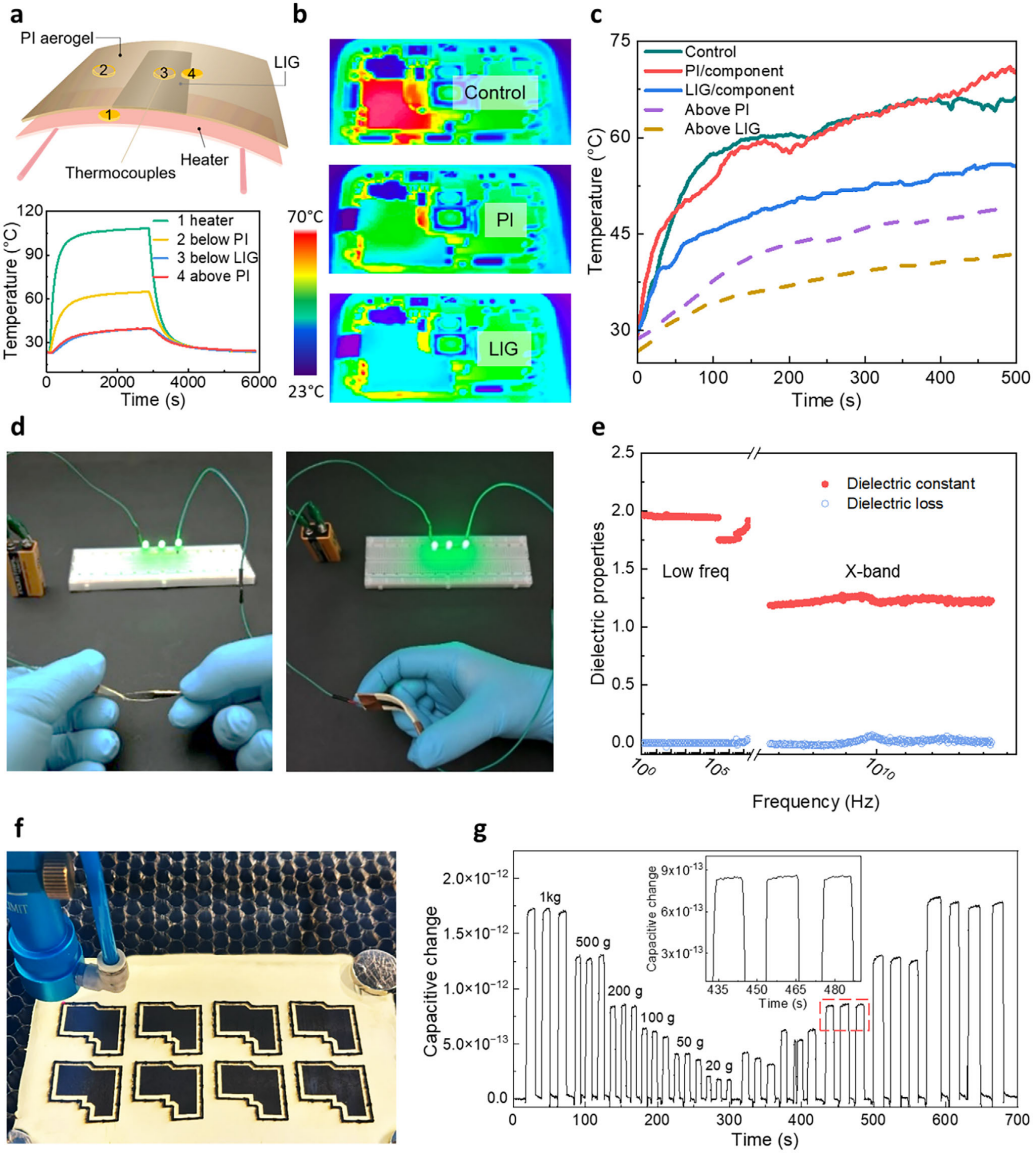
Electrical and thermal management with LIG‐polyimide aerogels (PI_DMAc_). a) Proof‐of‐concept of heat insulation and conduction with a single material. b) Thermal management of the heat on a 5G smartphone: IR images and c) temperature evolution. d) Mechanical distortion does not affect the electrical conductivity. e) Dielectric properties of the polyimide aerogel. f) Photo of laser writing and cutting. g) Capacitive response of a LIG‐PI aerogel pressure sensor; insert highlights the fast and complete recovery of the sensors.

Thermally, graphitization significantly increases the thermal conductivity.^[^ ^35^ ^]^ A simple proof‐of‐concept (**Figure** **4**a; Figure S15, Supporting Information) demonstrates the effectiveness of the laser‐patterned material as a heat sink: a much lower temperature is maintained on the LIG layer (position 3) compared to the unmodified PI substrate (position 2), even though both were maintained at the same distance from the heat source. In a second demonstration of thermal management, the application was tested on a smartphone (Figure 4b,c; Figure S16, Supporting Information), which combined heat conductors (LIG) and thermal insulators (PI aerogel substrate) to spread heat in two dimensions. This configuration is already standard in highly integrated microelectronics.^[^ ^36^ ^]^ Using a polyimide aerogel shield decreased the top surface temperature from 65 to 45 °C after 8 min of operation. Still, the component's temperature continued to rise to 70 °C because the heat was trapped beneath the cover (Figure 4c). In contrast, a shield with a laser‐induced graphitization layer on the polyimide aerogel reduced the surface temperature to 37 °C and the component's temperature to 52 °C, thanks to the spatially controlled conductive layer on the thermal insulator. This single material provided both insulation and heat dissipation, effectively reducing both internal and surface temperatures.

Electrically, the graphene layer exhibits very high and stable conductivity, even when the hybrid material undergoes physical distortion (Figure 4d). The polyimide aerogel substrate has excellent dielectric properties, with a low dielectric constant (ɛ_r_, 1.1–2.0) and loss (tan δ <0.2) across a wide frequency range (0.1 Hz to 12 GHz), covering radio wave to sub‐microwave regions, suitable for radar and satellite communications (Figure 4e). The combination of LIG patterning with flexible and machinable polyimide aerogels enables unrestricted design possibilities and rapid large‐scale production by simply adjusting the laser scanning power (Figure 4f; Table S4 and Movie S3, Supporting Information). Additionally, a LIG‐PI aerogel pressure sensor demonstrated impressive signal detection (Figure 4g; Figure S17, Supporting Information). The flexible (Figure S10a, Supporting Information) and low dielectric (Figure 4e) polyimide aerogel substrate, combined with the highly conductive graphene layer (Figure 3d,h), ensured high‐precision detection of small changes in compressive stress and strain (Figure 4g). The fast structural recovery of the polyimide aerogel, combined with LIG, allows for precise measurements across a wide range of loads (from 0.020 to 1.000 kg, corresponding to 0.87–43.60 mN mm^−2^, Table S5, Supporting Information) without compromising accuracy, providing a single “all‐in‐one” solution that eliminates the need for additional materials.

### 2.5 All‐In‐One Design of Patch Antenna

**Figure 5:**
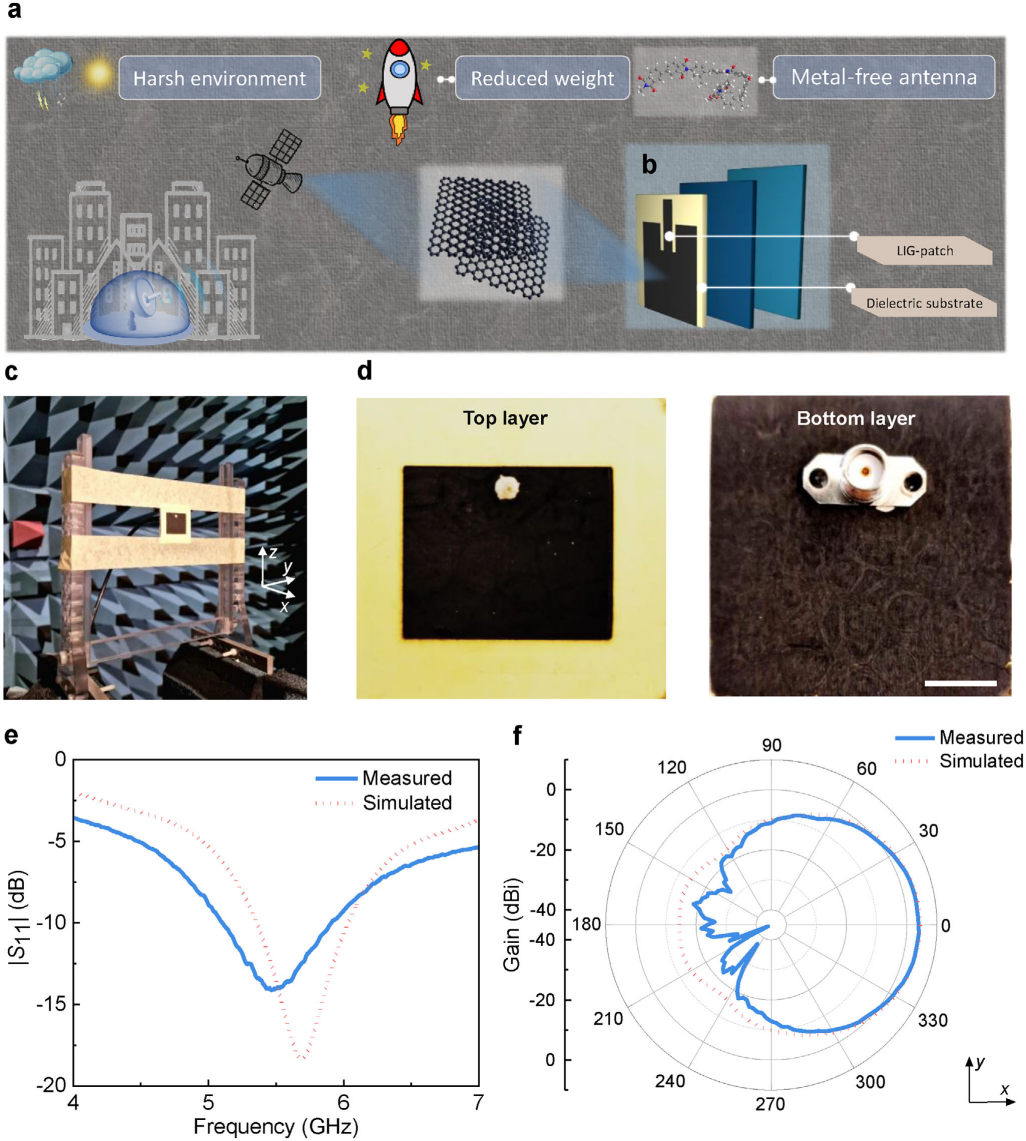
Design and characterization of polyimide aerogel‐LIG patch antenna. a) Illustration of the application cases of LIG‐patch/dielectric aerogel substrate in satellites and base stations, as well as the harsh service environments and demands they encounter during operation. b) The construction of the patch antenna. c) Characterization of the patch antenna in an electromagnetic anechoic chamber. d) The top and bottom layers of the fabricated patch antenna (scale bar 10 mm), using 3‐mm polyimide aerogel as the dielectric substrate and LIG as the conductive pattern. e) Reflection coefficients |*S*_11_| as a function of the frequency. f) The radiation pattern of the patch antenna at 5.5 GHz.

Moreover, owing to their low dielectric permittivity combined with excellent mechanical properties, polyimide aerogels hold significant promise as enabling technology for antenna systems.^[^ ^7^, ^37^ ^]^ Targeting applications in 5G, satellite, and aircraft communications (**Figure** **5**a), a patch antenna was designed (Figure S18, Supporting Information). Both the top and bottom conductive layers comprise LIG polyimide aerogel instead of conventional copper or gold patterns obtained *via* electron beam evaporation (Figure 5b). The performance benefits from the optimal sheet resistivity of the LIG layer of 6.5 Ωsq^−1^ and the low dielectric constant of the polyimide aerogel substrate of 1.08. As a patch antenna, the top conductive layer acts as a patch, while the bottom conductive layer acts as a ground plane. The antenna substrate is made of polyimide aerogel, and its thickness is adjusted to 3 mm through polishing. The patch antenna features a round hole through the top and bottom conductive layers for mounting a SubMiniature version A (SMA) connector (Figure S18, Supporting Information). Laser scanning was employed to precisely create the top and bottom conductive layers of the patch antenna (Figure 5d). The achieved patch antenna has a mass of only 0.89 g and was characterized in an electromagnetic anechoic chamber (Figure 5c). The reflection coefficient |*S*_11_|, which represents the ratio of the reflected wave amplitude to the incident wave amplitude at the antenna port,^[^ ^37^ ^]^ was measured to be −14.0 dB at an operating frequency of 5.5 GHz, with a −10 dB bandwidth of 0.8 GHz, i.e., from 5.11 to 5.91 GHz (Figure 5e). The measured 2D radiation pattern (Figure 5f) shows a peak gain of 3.9 dBi, aligning closely with the simulated results. Deviations between the measured and simulated values are likely due to minor deviations in the fabricated patch antenna, such as slight processing errors in the LIG layer or uneven thickness of the polyimide aerogel substrate. The simulated 3D radiation pattern is shown in Figure S19 (Supporting Information).

## 3 Conclusion

In summary, the composition and arrangement of polyimide chains containing flexible, high free‐volume (DMBZ) segments, together with the varied solvent‐polymer interactions in different solvent systems, led to the formation of polyimide aerogels with hierarchical and micro‐spherical porous structures. The resulting aerogels are flexible, hydrophobic, and exhibit excellent dimensional stability at elevated temperatures. They maintain low thermal conductivity even after prolonged exposure to high temperatures and possess a low dielectric constant and loss, making them ideal substrates for laser scribing. Through laser‐induced graphitization, the aerogels were seamlessly patterned with a continuous and highly conductive graphene layer. The integration of superior thermal and electrical conductivity on a flexible polyimide insulator opens a wide range of applications, including thermal and electrical management, high‐precision pressure sensors, and lightweight patch antennas.

## 4 Experimental Section

### Materials

4, 4'‐oxydianiline (ODA, 97%) and biphenyl‐3, 3', 4, 4'‐tetracarboxylic dianhydride (BPDA, 98%) were provided by Sigma–Aldrich. m‐tolidine (DMBZ, >98%) was obtained from TCI chemicals. The imidization agents: triethylamine (≥99%) was purchased from Sigma–Aldrich, and acetic anhydride (≥98%) was bought from. The crosslinker 1, 3, 5‐benzene tricarboxylic acid (BTC, 98%) was purchased from Sigma–Aldrich. N, N‐Dimethylacetamide (DMAc, 99%, Chemie Brunschwig AG) and N‐methyl‐2‐pyrrolidone (NMP, 98%, Chemie Brunschwig AG) were used as solvents for poly (amic acid) synthesis and sol‐gel processing. Ethanol (95%, with 5% isopropanol, Alcosuisse) was used for the solvent exchange prior to supercritical CO_2_ drying. All reagents were used as received without any further purification.

### Preparation of Polyimide Aerogels

In this work, polyimide aerogels were prepared based on previous work.^[^ ^21^, ^38^ ^]^ The BPDA‐ODA/DMBZ in DMAc solvent was selected here as an example for the demonstration of the synthetic process. In detail, 1.1373 g ODA was added in DMAc, and after dissolving completely, 3.2343 g BPDA was added, stirring for ≈5 min. Then, 1.2057 g DMBZ was added inside the suspension for another 30 min to get a homogeneous poly (amic acid) (PAA) oligomer solution. Premixed 8.313 mL acetic anhydride and 12.257 mL triethylamine were added to the PAA solution for ≈3 min. In the end, 0.0649 g BTC was added to the system. In total, the volume of DMAc is 80 mL, where the concentration of PAA is 7 wt.%. Then, the aged polyimide gels (for 12 h at ambient temperature) were stepwise solvent exchanged into ethanol (5% isopropanol) for supercritical CO_2_ drying (SCF extractor, Separex) at 120 bar and 50 °C. In the case of ambient pressure drying, the wet gels, after solvent exchange into ethanol, were put in a 65 °C oven.

### Laser Treatment

Polyimide aerogel substrates were directly scribed using a commercial 10.6 µm CO_2_ laser engraver (Speedy 400, Trotec). A laser power of 3.9 W, a scanning speed of 125 mm s^−1^, and an image density of 600 pulses per inch were used for the laser treatment. A 2.5'' lens was used, and the defocus was 5 mm (the resulting beam diameter was 0.4 mm). Laser power measurements were performed using a laser power meter (HLP‐200, NANJING STARTNOW OPTO ELECTRONICS CO., LIMITED), and the energy fluence was subsequently calculated based on the measured power and the approximated beam area (radius ≈400 µm, determined via optical measurements). The calculated energy fluence ranged from 6.5 to 8.5 J cm^−2^, which is consistent with the reported thresholds of 5 to 8 J cm^−2^ for laser engraving of polyimide films, as documented by Abdulhafez et al.^[^ ^39^ ^]^ However, due to differences in the laser parameter set employed in this study, including a defocused z‐position of −5 mm, the use of a 2.5″ lens, and a lower laser engraving speed of 125 mm s^−1^ compared to 500 mm s^−1^.

### Thermal Management

The infrared images in the high‐temperature range were recorded using TH 3102 MR (NEC‐San‐ei, Japan) equipped with a Stirling‐cooled HgCdTe detector (−50–500 °C), with a temperature sensitivity of 0.08 at 30 °C and an accuracy of ± 0.5 °C. The emission was set to 1. Thermal images were analyzed on a PicWin‐IRIS system (version 7.3). Thermal couples (TASi TA612C K/J thermometer) were used to record the temperature of the cellphone. All the temperature and thermal images were recorded when the cellphone (Oneplus 6T) was recording a 4K 60fps HDR video.

### Pressure Sensing

To demonstrate the proof‐of‐concept pressure sensor, the polyimide aerogel not only as a substrate but also as a dielectric within a parallel plate structure, enabling a capacitive sensing principle was used. Thus, 1.5 mm thick polyimide aerogels from both the upper and lower sides with 20 × 20 mm^2^ electrodes was laser‐engraved. By applying pressure, for example, by loading the sensor structure with different weights (Figure S15, Supporting Information), a change in capacity is measured in accordance with the Equation S1 (Supporting Information).

### Design and Characterization of Polyimide Aerogel‐LIG Patch Antenna

The patch antenna, consisting of a 3 mm polyimide aerogel substrate and produced by LIG, was connected with an RF coaxial SMA connector through conductive silver epoxy (CW2460, Chemtronics). The antenna was numerically simulated using the electromagnetic full‐wave simulation solver CST Microwave Studio. The antenna's reflection coefficient was measured by a vector network analyzer (VNA, 8720D HP). For the far‐field measurements of antennas, an experimental setup was built in an electromagnetic anechoic chamber. A quad‐ridge horn antenna (QH400, MVG Industries) was used as a reference antenna and fixed at 2.1 m from the antenna under test for radiation pattern measurement. The polarization direction of the reference antenna was set to coincide with the orientation of the patch antenna.

Details of the characterization, measurements, and demonstrations (thermal management, pressure sensing, and patch antenna), along with the parameters, equations, and analysis for small‐angle X‐ray scattering, were provided in the Supporting Information.

## Conflict of Interest

The authors declare no conflict of interest.

## Supporting information



Supporting Information

Supplemental Movie 1

Supplemental Movie 2

Supplemental Movie 3

## Data Availability

The data that support the findings of this study are available from the corresponding author upon reasonable request.
