# Supplementary material for: Designing Stable Graphitic Networks on Ultra‐Porous Polyimide Aerogels via Solvent‐Guided Structuring
Source: Small. 2025 Dec 12;22(6):e05776. doi: 10.1002/smll.202505776 (PMC12837349; doi:10.1002/smll.202505776)
Supplement: Supplementary file 1 — Supporting Information [file SMLL-22-e05776-s001.docx]

Supplementary Materials for

**Designing Stable Graphitic Networks on Ultra-porous Polyimide Aerogels via Solvent-Guided Structuring**

Tingting Wu ^1,2,†^, Mengmeng Li ^1,†^, Mingxiang Gao ^3^, Christopher H. Dreimol ^4, 5^, Ekaterina Filimonova ^1^, Qin Li ^6^, Yunhong Wang ^6^, Dimitrios Sapalidis ^7^, Michal Ganobjak ^1^, Yanfang Wei ^1^, Joshua Yip ^1^, Bruno F. B. Silva ^7, 8, 9^, Anja K. Skrivervik ^3^, Wim J. Malfait ^1^, Shanyu Zhao *^, 1^

1 T. Wu, M. Li, E. Filimonova, M. Ganobjak, Y. Wie, J. Yip, W. J. Malfait, S. Zhao
Laboratory for Building Energy Materials and Components, Empa, Swiss Federal Laboratories for Materials Science and Technology, Überlandstrasse 129, 8600 Dübendorf, Switzerland.

^†^ These authors contributed equally: T. Wu, and M. Li
E-mail: [shanyu.zhao@empa.ch](mailto:shanyu.zhao@empa.ch)

2 T. Wu
Institute of Sustainability for Chemicals, Energy and Environment (ISCE2), Agency for Science, Technology and Research (A*STAR), 1 Pesek Road, Jurong Island 627833, Singapore

3 M. Gao, A. K. Skrivervik
Microwaves and Antennas Group, Institute of Electrical and Micro Engineering, École Polytechnique Fédérale de Lausanne, Lausanne, Switzerland

4 C. H. Dreimol
Wood Materials Science, Institute for Building Materials, ETH Zürich, 8093 Zürich, Switzerland

5 C. H. Dreimol
Cellulose & Wood Materials Laboratory, Empa, 8600 Dübendorf, Switzerland

6 Q. Li, Y. Wang
IBIH Advanced Material Co., Ltd, Lingang Economic And Technological Development Zone, 061108, Cangzhou, China

7 D. Sapalidis, B.F.B. Silva
Center for X-ray Analytics, Empa, Swiss Federal Laboratories for Materials Science and Technology, Lerchenfeldstrasse 5, 9014 St. Gallen, Switzerland

8 B. F. B. Silva
Laboratory for Biointerfaces, Swiss Federal Laboratories for Materials Science and Technology, Lerchenfeldstrasse 5, 9014 St. Gallen, Switzerland

9 B. F. B. Silva
Laboratory for Biomimetic Membranes and Textiles, Empa, Swiss Federal Laboratories for Materials Science and Technology, Lerchenfeldstrasse 5, 9014 St. Gallen, Switzerland

**This PDF file includes:**

**Supplementary Note:**

Characterization and Measurements

Small-Angle X-ray Scattering analysis (Zimm Analysis, Guinier-Porod model, Teubner-Strey model)

**Supplementary Figures**

**Figure S1.** The SEM images of polyimide aerogels from V_NMP_/V_DMAc_=3:7, V_NMP_/V_DMAc_=1:9, dried by SCD, and from pure DMAc dried by APD.

**Figure S2.** The SEM images of polyimide aerogels from pure DMAc, solvent exchanged into hexane and heptane, instead of conventional ethanol, dried by APD.

**Figure S3.** The lower magnification SEM images of polyimide aerogels prepared under different conditions.

**Figure S4.** Zimm plot of the aerogels in the different solvents and the corresponding linear fitting.

**Figure S5.** SAXS patterns of the aerogels fitted using the Teubner-Strey model.

**Figure S6.** Gels formation timelines.

**Figure S7.** Independent Gradient Model based on Hirshfeld partition (IGMH) results of the interaction between defined polyimide structure and NMP.

**Figure S8.** Compressive Young’s modulus and specific Young’s modulus.

**Figure S9.** The Young’s modulus of polyimide aerogels from 3-point bending test.

**Figure S10.** Mechanical properties of the polyimide aerogels (a) flexibility of the PI_DMAc_ aerogel, (b) stress-strain curves of aerogels under various conditions.

**Figure S11.** (a) N_2_ isotherms and (b) specific surface area and pore volume of all the polyimide aerogels under various preparation conditions.

**Figure S12.** The N_2_ isotherms of all the polyimide aerogels dried from different solvents.

**Figure S13.** Outer surface structures of the samples prepared under different conditions: (a) photographs of samples prepared from NMP, 50NMP_50DMAc, and DMAc solvents, and SEM images at different magnifications of (b1–3) NMP, (c1–3) 50NMP_50DMAc, and (d1–3) DMAc samples.

**Figure S14.** IR images of six aerogel cylinders (thickness: 10 mm) from different recipes at 0 min and 67 min; temperature evolution of the upper surface of different aerogels with time.

**Figure S15.** The setup for thermal management of LIG-polyimide aerogels.

**Figure S16.** The setup for thermal management of LIG-polyimide aerogels on a 5G smartphone.

**Figure S17.** The setup (a) and schematic representation (b) of LIG-polyimide aerogels as a pressure sensor.

**Figure S18.** Proposed patch antenna design.

**Figure S19.** Simulated 3D radiation pattern of the patch antenna.

**Supplementary Tables**

**Table S1.** SAXS analysis data for the three polyimide samples using the Generalized Guinier-Porod model.

**Table S2.** SAXS analysis data for the three polyimide samples using the Teubner-Strey model.

**Table S3.** Comparison of the reported polyimide aerogels: dielectric, thermal conductivity, and density.

**Table S4.** Parameters used for the optimization of the LIG on polyimide aerogels

**Table S5.** Calculated value of mass in kg and pressure in mN/mm^2^ for pressure sensor demonstration.

**Other Supplementary Materials:**

Movies S1 to S3

**Characterization and Measurements**

**Density functional theory (DFT) simulation.** All the calculations of the model compounds studied in this work were performed using the Gaussian 16 software package[1]. The optimal conformations for polyimide (BPDA-ODA-DMBZ), DMAc, and NMP were calculated by DFT at the B3LYP/6-31G (d, p) level with the D3(BJ) dispersion. In addition, the composite structures were calculated by DFT at the B3LYP-D3/6-31G (d, p) level for the atoms in geometry optimizations. Then, the IGMH analysis was calculated based on the optimal structure with electronic wave function information using the Multiwfn 3.7(dev) program[2]. Vibrational frequency analyses at the same level of theory were performed to characterize stationary points as local minima without any imaginary frequencies. The single-point energy calculations were carried out using the 6-311++g(d,p) basis set[3] to provide better energy correction.

**Small-Angle X-ray Scattering (SAXS).** The SAXS measurements were conducted using a Bruker Nanostar instrument (Bruker AXS GmbH, Karlsruhe, Germany) equipped with a pinhole collimation system and a micro-focused Cu X-ray source (Incoatec), providing Cu K_α_ radiation with a wavelength of *λ*=1.5406 Å and a beam spot size of approximately 400 μm. The system featured a 2D MikroGap technology-based detector (VÅNTEC-2000) with 2048 × 2048 pixels, each 68 × 68 μm^2^ in size, offering a resolvable *q*-range between 0.07 and 2.10 nm^-1^ at a sample-to-detector distance of 107 cm. The system also included a semi-transparent beam stop for scattering curve normalization relative to the direct beam intensity. SAXS intensity data are reported in arbitrary units as a function of *q*, the momentum transferred during a scattering event (*q = (4π/λ) × sin(2θ/2),* where *2θ* is the scattering angle).

Measurements were conducted under vacuum conditions (∼0.01 mbar) to minimize background scattering from the air. Kapton tape was used to mount the samples, ensuring that measurement points were on the aerogel volume without Kapton interference. The exposure time for the aerogel samples was 100 seconds. Background scattering from the sample environment was used for the free-standing aerogel samples, and all patterns were transmission corrected using direct beam measurement. Data processing and analysis were conducted with SASfit Toolbox[4] and Python code.

**Microstructural analysis.** SEM images were acquired on a FEI Nova NanoSEM 230 at an accelerating voltage of 5 kV and a working distance of ~5 mm. 15 nm of Pt (measured with a flat piezo detector) was coated to avoid charging prior to the measurement. Nitrogen sorption analysis was carried out on a nitrogen sorption analyzer (TriFlex, Micromeritics) after prior degassing for 15 h at 80 °C and 0.02 mbar. The specific surface areas (S_BET_, uncertainty ~50 m^2^ g^-1^) were calculated by the Brunauer-Emmett-Teller (BET) method.

**Raman spectroscopy.** Raman spectroscopy was conducted using a confocal Raman microscope (Renishaw InVia), equipped with a 532 nm laser, a 20× objective (Zeiss), and an 1800 mm^-1^ grating. The integral exposure time for single-point measurements was three seconds, with 10 accumulations and a laser power of 2 mW over a spectral range of 1220–2790 cm^-1^. Analogous parameters were employed for the Raman mapping of the laser-graphitized surface structure. However, only a single accumulation was measured. The mapping area was 50 × 50 µm^2^, with a step width of 250 nm (map image acquisition mode). Subsequently, the data were subjected to baseline correction and a cosmic ray filter using the software Wire 4.4 (Renishaw, UK). The data were analyzed and plotted using OriginPro 2019 (version 9.6.0.172, OriginLab Corporation, US). The software Cytospec (v. 2.00.07), integrated into MatLab, was employed for chemical map images of the intensity ratio of I_D_/I_G_. Here, the intensity range of 1340–1360 cm^-1^ for the D-peak and 1570–1590 cm^-1^ for the G-peak was used.

**Thermal stability.** Thermogravimetric analysis was conducted on a Netzsch TG 209F1 Thermogravimetric Analyzer operating at 10 K min^-1^ in a reconstituted air atmosphere. The volumetric shrinkages of polyimide aerogels were evaluated after exposure to 200 °C and 300 °C for 1 hour. The infrared images of the aerogel cylinders and phoenix aerogel pattern on the thermal stage were recorded using a Testo 880-1 (measuring range 0–350 °C).

**Hydrophobicity.** The surface wettability of samples was evaluated by measuring the water contact angle using a Contact Angle System OCA (Dataphysics TBU 90E, Germany) combined with a high-speed camera. The volume of the water droplet was 10 μL, and the tip was a precision stainless steel tip (Gauge 32, EFD).

**Thermal conductivity.** Thermal conductivities were determined from square plate aerogels (around 50 × 50 × 8 mm^3^) using a custom-built guarded hot plate device (guarded zone: 50 × 50 mm^2^, measuring zone: 25 × 25 mm^2^) designed for small samples of low thermal conductivity materials with a 10 °C temperature difference[5].

**Dielectric properties.** The waveguide transmission technique was employed to test the dielectric properties across four frequency ranges. For low-frequency measurements (10^-1^ to 10^6^ Hz), the sample was measured using a parallel plate capacitor with a Novocontrol impedance Alpha Analyzer combined with a Quatro temperature controller. In the gigahertz frequency range, tests were performed using the waveguide method of a vector network analyzer (VNA, Agilent PNA N5244A). The sizes of the tested samples were 22.86 mm × 10.16 mm (length × width) for the frequency range of 8.2–12.4 GHz (X-band).

**Electrical property characterization.** 40 × 40 mm² squares were laser-engraved onto 50 × 50 mm² polyimide samples. Sheet resistance was measured using a four-point probe system (SD-800, NAGY), while electrical resistivity was determined with a source measure unit (Model 2450, Keithley Instruments, USA). Sheet resistance mapping was performed using the EddyCus® TF map 2525SR automated mapping system, which was calibrated with 50 × 50 mm² reference samples based on NIST standards.

**Demonstrations**

**Thermal management.** The infrared images in the high-temperature range were recorded using TH 3102 MR (NEC-San-ei, Japan) equipped with a Stirling-cooled HgCdTe detector (-50 to 500 °C), with a temperature sensitivity of 0.08 at 30 °C and an accuracy of ± 0.5 °C. The emission was set to 1. Thermal images were analyzed on a PicWin-IRIS system (version 7.3). Thermal couples (TASi TA612C K/J thermometer) were used to record the temperature of the cellphone. All the temperature and thermal images were recorded when the cellphone (Oneplus 6T) was recording a 4K 60fps HDR video.

1. The thermal management test setup is shown in Fig. S13. The DMAc_SCD sample was laser engraved to form a graphene strip (5 mm width × 20 mm length) on a polyimide strip sample (10 mm width × 25 mm length). At one end of the sample, it was in close contact with a ceramic heater (40 × 40 × 2 mm^3^, 24 V, 21–27 Ω), which was connected to a power supply (BEHA NG 303 Labornetzgerät 0–32 V). Four thermocouples were placed on the heater (①), the graphene layer (②), the same side of the polymer (③), and the opposite side on the top of the polymer (④).
2. The thermal management test on the smartphone is shown in Fig. S14. First, the hottest element on the cellphone (Oneplus 6T) was identified using a thermal imager. Then, the manufactured cover (Fig. 4f) was placed over the component. Two thermocouples were placed between the component and the polymer cover (in contact with the graphene layer when the LIG sample was used and in contact with the polymer when the insulator was used) and on the top of the polymer cover.

**Pressure sensing**. To demonstrate the proof-of-concept pressure sensor, we used the polyimide aerogel not only as a substrate but also as a dielectric within a parallel plate structure, enabling a capacitive sensing principle. Thus, we laser-engraved 1.5 mm thick polyimide aerogels from both the upper and lower sides with 20 × 20 mm^2^ electrodes. By applying pressure, for example, by loading the sensor structure with different weights (Fig. S15), a change in capacity is measured well in accordance with the following equation:

$C\left( F \right)=\varepsilon_{0}\varepsilon_{R}\frac{A}{d}\frac{AE}{AE-F}$ (S1)

in which A is the electrode area, E is the Young modulus, d is the distance between plates, and F is the force[6]. The capacitance changes were measured by probing with an LCR meter (Agilent E4980A precision, USA), recording at 100 kHz frequency and 1 V. Prior to each measurement, the cables were calibrated for short and open circuits.

Design and characterization of polyimide aerogel-LIG patch antenna. The patch antenna, consisting of a 3 mm polyimide aerogel substrate and produced by LIG, was connected with an RF coaxial SubMiniature version A (SMA) connector through conductive silver epoxy (CW2460, Chemtronics). The antenna is numerically simulated using the electromagnetic full-wave simulation solver CST Microwave Studio. The antenna’s reflection coefficient was measured by a vector network analyzer (VNA, 8720D HP). For the far-field measurements of antennas, an experimental setup was built in an electromagnetic anechoic chamber. A quad-ridge horn antenna (QH400, MVG Industries) was used as a reference antenna and fixed at 2.1 m from the antenna under test for radiation pattern measurement. The polarization direction of the reference antenna was set to coincide with the orientation of the patch antenna.

**
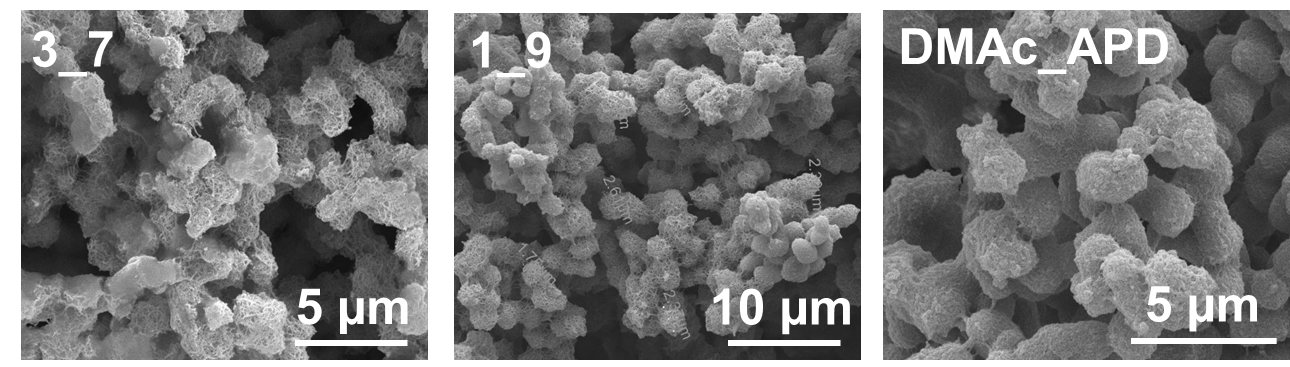
**

**Figure S1.** The SEM images of polyimide aerogels from V_NMP_/V_DMAc_=3:7, V_NMP_/V_DMAc_=1:9, dried by SCD, and from pure DMAc dried by APD.


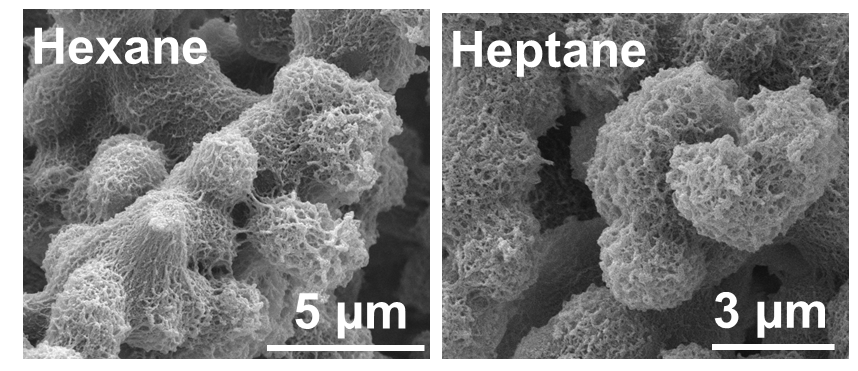


**Figure S2.** The SEM images of polyimide aerogels from pure DMAc, solvent exchanged into hexane and heptane, instead of conventional ethanol, dried by APD.


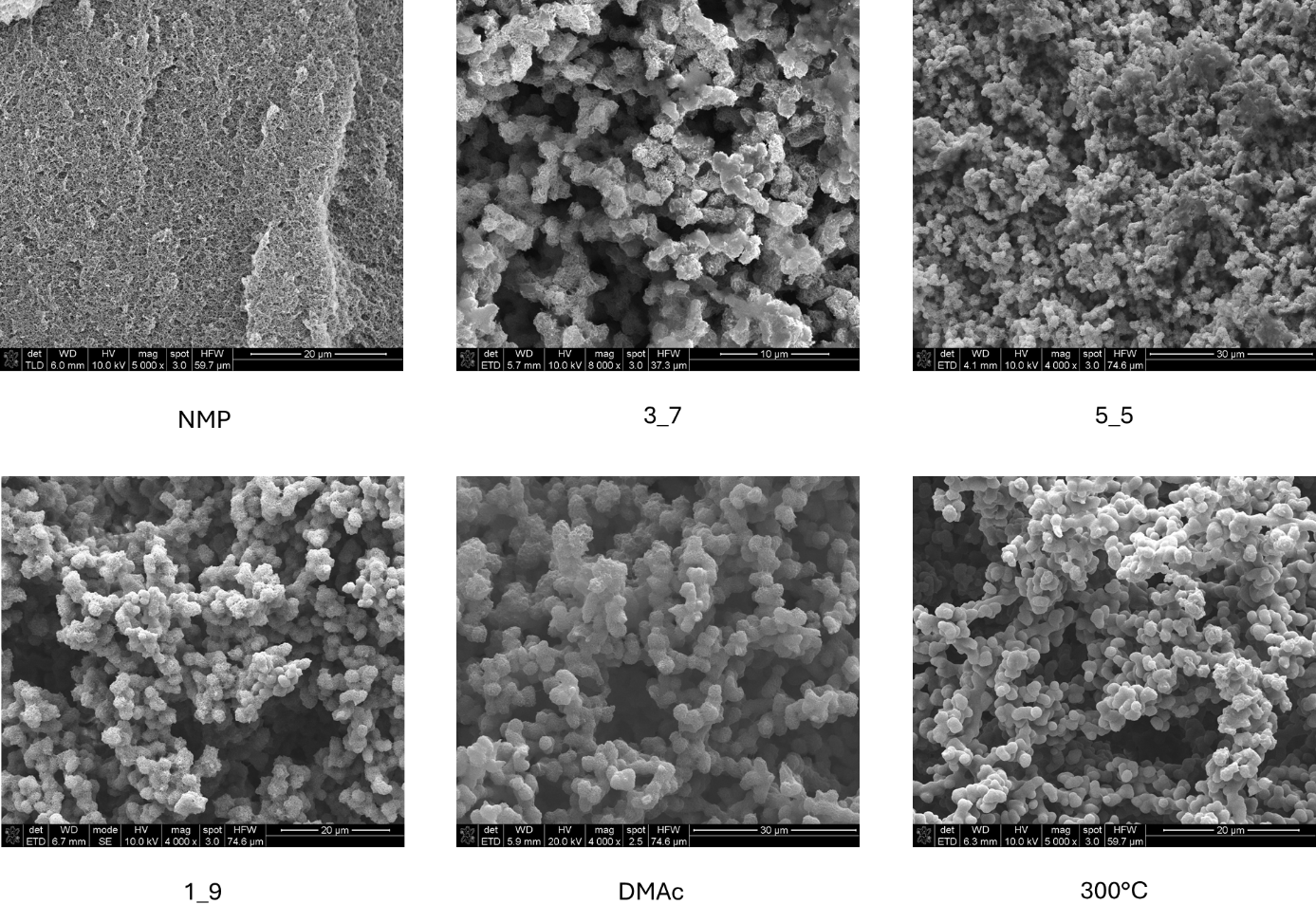


**Figure S3.** The lower magnification SEM images of polyimide aerogels prepared under different conditions.

**Small-Angle X-ray Scattering**

**Zimm Analysis**

Absolute intensity calibration was performed using distilled water standard in a Hilgenberg quartz capillary of 1.5 mm width with a 0.01 mm wall thickness. The measurement time was 1 hour in the same configuration and conditions as the aerogel measurements. The water SAXS pattern was transmission corrected using direct beam measurement and the capillary’s scattering contribution was subtracted. The absolute intensity calibration constant is given by the following equations[7]:

$C\left( \lambda_{Cu-K_{\alpha}} \right)=\frac{a_{H_{2}O}}{d_{H_{2}O}\left( \frac{d\Sigma}{d\Omega} \right)_{H_{2}O}}=8.452$ (S2)

Here the constant $a_{H_{2}O}=0.02064$ is obtained from the linear fitting of the time-normalized water scattering intensity (after background subtraction); $d_{H_{2}O}=0.148 cm$ is the water thickness illuminated by the beam; and $\left( \frac{d\Sigma}{d\Omega} \right)_{H_{2}O}={0.0165 cm}^{-1}$ is the water scattering cross-section.

The absolute scattering intensity of the aerogels is calculated using the calibration constant in combination with the sample’s thickness (*d_aerogel_*=0.6 cm) and the exposure time (*t*=100 s):

$\left( \frac{d\Sigma}{d\Omega} \right)_{aerogels}=\frac{I_{exp}^{aerogel}}{d_{aerogel} C\left( \lambda_{Cu-K_{\alpha}} \right)t}$(S3)

Zimm plots are typically used to estimate the molecular weight (*M*) and radius of gyration (*R_g_*) of polymer chains. In the low q region, the plot presents a linear behavior as can be seen in Figure S4. The following formula is valid for the low q region and low concentration[8]:

$\frac{1}{\frac{d\Sigma}{d\Omega}\left( q \right)}\approx\frac{N_{A}\rho^{2}}{\left( \Delta\eta\right)^{2}cM}\left( \frac{q^{2}R_{g}^{2}}{3} \right)$ (S4)

where *ρ* is the bulk density of the polymer, $\Delta\eta$ the scattering length density of the sample, *c* the concentration, and *N_A_* the Avogadro constant. The values of *R_g_* as obtained from fitting eq. S4 to the data are: 16.8 nm for the NMP sample, 44.5 nm for DMAc/NMP, and 45.8 nm for DMAc. These values, especially for the DMAc/NMP and DMAc solvents, are significantly larger than the values obtained using the generalized Guinier-Porod model with mass fractal exponential cutoff structure factor (described below). The discrepancy is likely due to the existence of hierarchical structures, as inferred from the upturns in the low *q* region (especially visible in the Kratky plots of the DMAc/NMP and DMAc solvents), which results in deviations of the *R_g_* obtained from the Zimm plots.


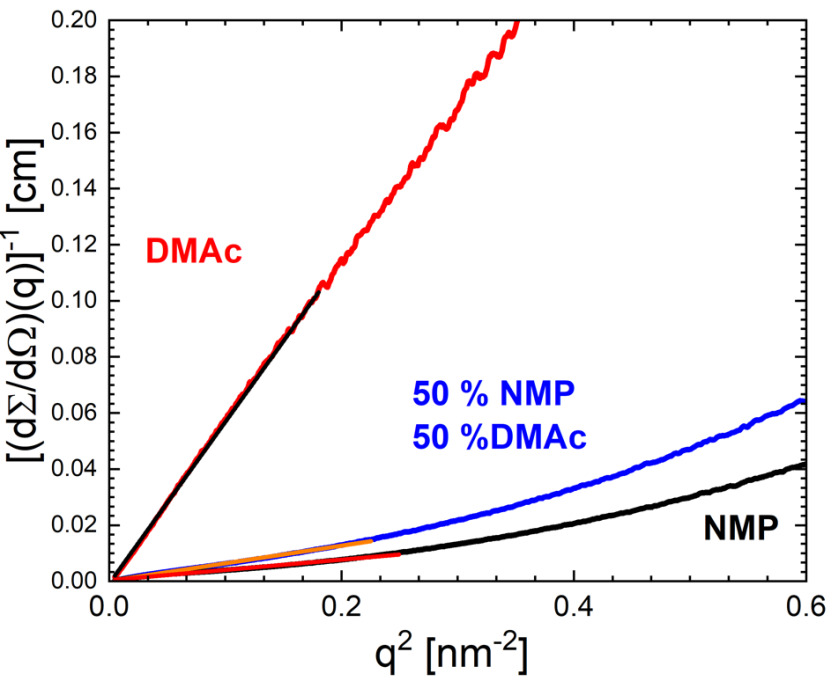


**Figure S4.** Zimm plot of the aerogels in the different solvents and the corresponding linear fitting.

**Generalized Guinier-Porod model fitting**

The polyamic acid skeleton consists of randomly oriented nanofibers that may form clusters, as evident in the SEM images. Typically, nanofibers exhibit a power law decay characterized by a *q^1^* slope, indicative of their linear, rigid structure, extending to relatively high *q*. However, in this case, the presence of a correlation peak in the intermediate *q* region and the transition to a *q^-4^* slope at higher *q* values suggest the presence of smaller structural features approximately 2-3 nm in size. It is not entirely clear what such features represent, but they could stem from voids between the fibers. Furthermore, the observed upturns in the low *q*-region, particularly noticeable in the DMAc and DMAc/NMP samples, suggest that mass fractals formed by nanofibers also significantly contribute to the scattering intensity. Due to the similarity in size between primary features (possibility voids) and secondary features (nanofibers’ cross-sections), the scattering profiles exhibit continuous curvatures that can obscure power-law regions. Therefore, to ensure objective data analysis, the scattering profiles were analyzed using the generalized Guinier-Porod unified model, which applies continuous analytical methods to fit the scattering profiles to multiple structural levels, each characterized by a power-law and a Guinier region. In our case, two Guinier regions were included:

$I\left( q \right)=\left\{ \begin{aligned} \frac{G_{2}}{q_{2}}\exp\left( -\frac{q^{2}R_{G2}^{2}}{3-s_{2}} \right) for q\leq q_{2} \\ \frac{G_{1}}{q_{1}}\exp\left( -\frac{q^{2}R_{G1}^{2}}{3-s_{1}} \right) for q_{2}\leq q\leq q_{1} \\ \frac{D}{q^{m}} for q>q_{1} \end{aligned} \right.$ (S5)

To obtain a continuous function in *I(q)* and its derivative, constraints are imposed on two chosen parameters, *q_2_* and *G_2_*, yielding the following result:

$$q_{2}=\sqrt{\frac{s_{1}-s_{2}}{\left( \frac{2}{3-s_{2}} \right)R_{g2}^{2}-\left( \frac{2}{3-s_{1}} \right)R_{g1}^{2}}}$$

$G_{1}=G_{2}\exp\left[ -q_{2}^{2} \left( \frac{R_{g1}^{2}}{3-s_{1}}-\frac{R_{g2}^{2}}{3-s_{2}} \right) \right]q_{2}^{(s_{2}-s_{1})}$ (S6)

$$q_{1}=\frac{1}{R_{g1}}\sqrt{\left( m-s_{1} \right)\frac{\left( 3-s_{1} \right)}{2}}$$

$$D=G_{1}\exp\left( \frac{q_{1}^{2}R_{g1}^{2}}{3-s_{1}} \right)q_{1}^{m-s_{1}}$$

The parameters are constrained such that *R_g2_>R_g1_*, *3>s_1_>s_2_*, and *m>s_1_*, ensuring that the functions and their derivatives exhibit strong monotonic decay. These constraints are necessary to achieve continuity in the function and its derivative at *q_1_* and *q_2_*.

Additionally, to account for the exponential behavior at low *q* due to mass fractals of the fiber aggregates or collapsed chains, a structure factor of mass fractal with exponential cutoff was considered. For a fractal aggregate, the analytical expression of the structure factor is given below:

$S\left( q \right)=C\left( qR_{g} \right)^{-D}$ (S7)

where *R_g_* is the radius of gyration of the individual scatterers (fibers) constituting the hierarchical structure, and *D* is the dimensionality of the fractal. The structure factor consists of two components: the power law *(qR_g_)^-D^* and the coefficient *C*, which depends on the cutoff-function *h(r/ξ)* where in our case (simple exponential):

$h\left( \frac{r}{\xi} \right)=\exp\left( -\frac{r}{\xi} \right)$ (S8)

The fitting results are presented in Table S1. A reduction of the nanofibers’ radius in DMAc compared to NMP is more visible through the fitting analysis. As previously discussed, all samples exhibit small features, which could be globular voids between the fibers. The average radius of these features decreases in DMAc down to 1.5 nm, while in NMP, they are slightly larger, up to 2.3 nm. The decrease in nanopore size could be attributed to the shrinkage of the close-to-spherical mass fractals observed in the NMP and NMP/DMAc samples into a 2D aggregate, as seen in the DMAc sample. This shrinkage could also explain the behavior of the polymer chains in DMAc during solvent evaporation, which may lead to greater densification, reducing the nanofibers’ radius more significantly compared to NMP polymeric fibers. Additionally, in a less effective solvent like DMAc, polymeric molecules may tend to aggregate more due to weaker solvation. This could provoke the formation of tighter, more compact nanofibers. On the other hand, in good solvents, the polymer coils may expand, so at the intermediate *q*-range, the slope tends to shift from *q*^-2^ (such as DMAc) to *q*^-5/3^ (such as NMP)[9]. The dimensionality of the fibers is close to 2, indicating they possess 2D cylindrical cross-sections.

Table S1. SAXS analysis data for the three polyimide samples using the Generalized Guinier-Porod model. The primary features might be related to the voids between the fibers (spherically shaped), while the secondary features correspond to the nanofibers’ cross-section. The size of the mass fractals is out of the *q*-range of SAXS. The dimensionality parameter for the first Guinier region *d_s_ = 3- s_2_ = 3 (s_2_=0)*, which is typical for cylindrical objects.

| Sample | Voids (Primary Features) | | | Fibers (Secondary Features) | | | | Mass Fractals | |
| --- | --- | --- | --- | --- | --- | --- | --- | --- | --- |
|  | m slope | R_g1_ (nm) | R_1_ (nm) | s_1_ | d_S_ | R_g2_ (nm) | R_2_ (nm) | D_m_ | L_m_ (nm) |
| in NMP | 4.17 | 2.32 | 3.00 | 1.07 | 1.93 | 14.64 | 20.70 | 2.73 | >400 |
| in 50 % NMP & 50 % DMAc | 4.26 | 1.87 | 2.42 | 1.36 | 1.64 | 10.14 | 14.34 | 2.85 | >400 |
| in DMAc | 4.33 | 1.48 | 1.91 | 1.22 | 1.78 | 8.32 | 11.77 | 1.91 | >400 |

The resulting fiber radii are reasonable comparing with the SEM images of the samples and with previous studies on polymeric aerogels[10, 11].

**Teubner-Strey model fitting inter-comparison**

The Teubner-Strey (TS) model[12] is frequently applied to model bicontinuous phases found in microemulsions and hydrogels. It facilitates the determination of domain size 𝑑 and correlation length 𝜉 using peak positions and the decay of scattering intensity. This phenomenological model is based on a Ginzburg-Landau expansion[13]. The analytical expression of the correlation peak observed in these systems is as follows:

$I\left( q \right)=\frac{1}{a_{2}+c_{1}q^{2}+c_{2}q^{4}} with a_{2}>0, c_{1}<0, c_{2}>0$(S9)

This model produces a single broad scattering peak, and a *q^-4^* decay at large *q*. The peak originates from the modulation in the corresponding space correlation function given by:

$\gamma\left( r \right)=\left( \frac{d}{2\pi r} \right)\exp\left( -\frac{r}{\xi} \right)\sin\left( \frac{2\pi r}{d} \right)$(S10)

with three free parameters: the characteristic length for positional correlation *ξ*, the characteristic domain size *d*, and the mean square scattering length density fluctuation, which is equal to *<η^2^> = φ(1-φ)Δn^2^* for sharp interfaces (such as polymeric fibers and voids) where φ is the porous volume fraction and *Δn* is the scattering contrast.


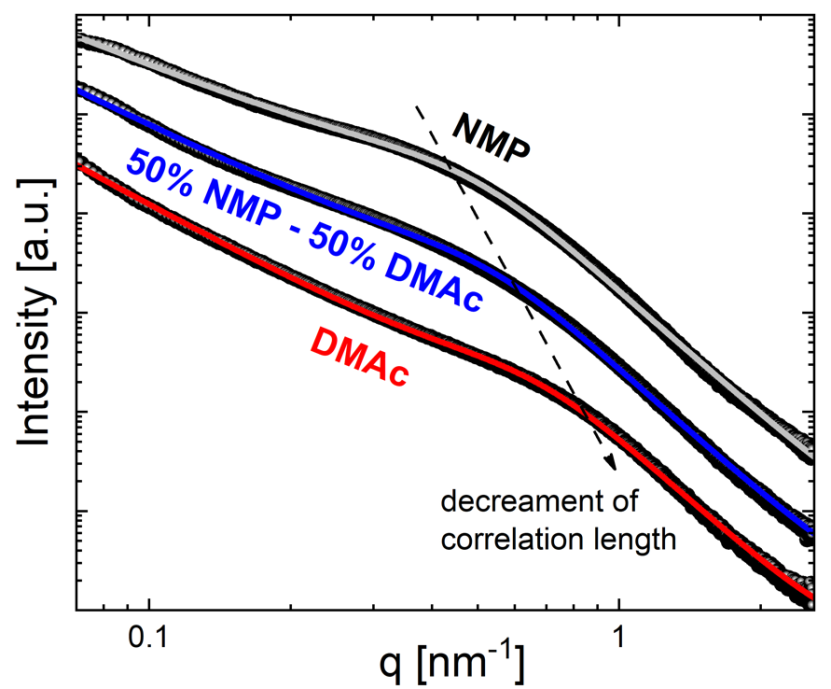


**Figure S5**. SAXS patterns of the aerogels fitted using the Teubner-Strey model.

To verify the validity of the generalized Guinier-Porod (GP) model, we also fitted the data using the TS model. Although the TS model reasonably explains the experimental data, its fitting quality was slightly inferior to that of the GP model, as indicated by lower *R^2^* values. Moreover, the GP model yields structural parameters that can be verified by SEM images, such as the shape of the fibers and the overall shape of the fractals. While we prefer to rely on the results obtained from the GP model, it is worth noting that the TS model provides relatively consistent results with the GP model. For instance, the correlation length 𝜉 and domain size 𝑑 from the TS model are similar to the radii of the primary and secondary features from the GP model. This consistency supports the reliability of the GP model analysis (Table S2).

**Table S2**. SAXS analysis data for the three polymeric samples using the Teubner-Strey model. The size of the mass fractals is out of the *q*-range of SAXS. For the mass fractals, the individual scatterers were chosen to match the size of the primary particles. The fitting results of correlation length and domain size are similar to those obtained from the generalized Guinier-Porod model.

| Sample | Voids (Primary Features) | | | Fibers (Secondary Features) | | | Mass Fractals | |
| --- | --- | --- | --- | --- | --- | --- | --- | --- |
|  | Guinier-Porod | | Teubner-Strey | Guinier-Porod | | Teubner-Strey | Teubner-Strey | |
|  | R_g1_ (nm) | R_1_ (nm) | Correlation length ξ | R_g2_ (nm) | R_2_ (nm) | Domain size d | D_m_ | L_m_ (nm) |
| in NMP | 2.32 | 3.00 | 2.98 | 14.64 | 20.70 | 16.36 | 1.94 | >400 |
| in 50 % NMP & 50 % DMAc | 1.87 | 2.42 | 2.79 | 10.14 | 14.34 | 14.68 | 2.30 | >400 |
| in DMAc | 1.48 | 1.91 | 2.28 | 8.32 | 11.77 | 11.37 | 2.55 | >400 |


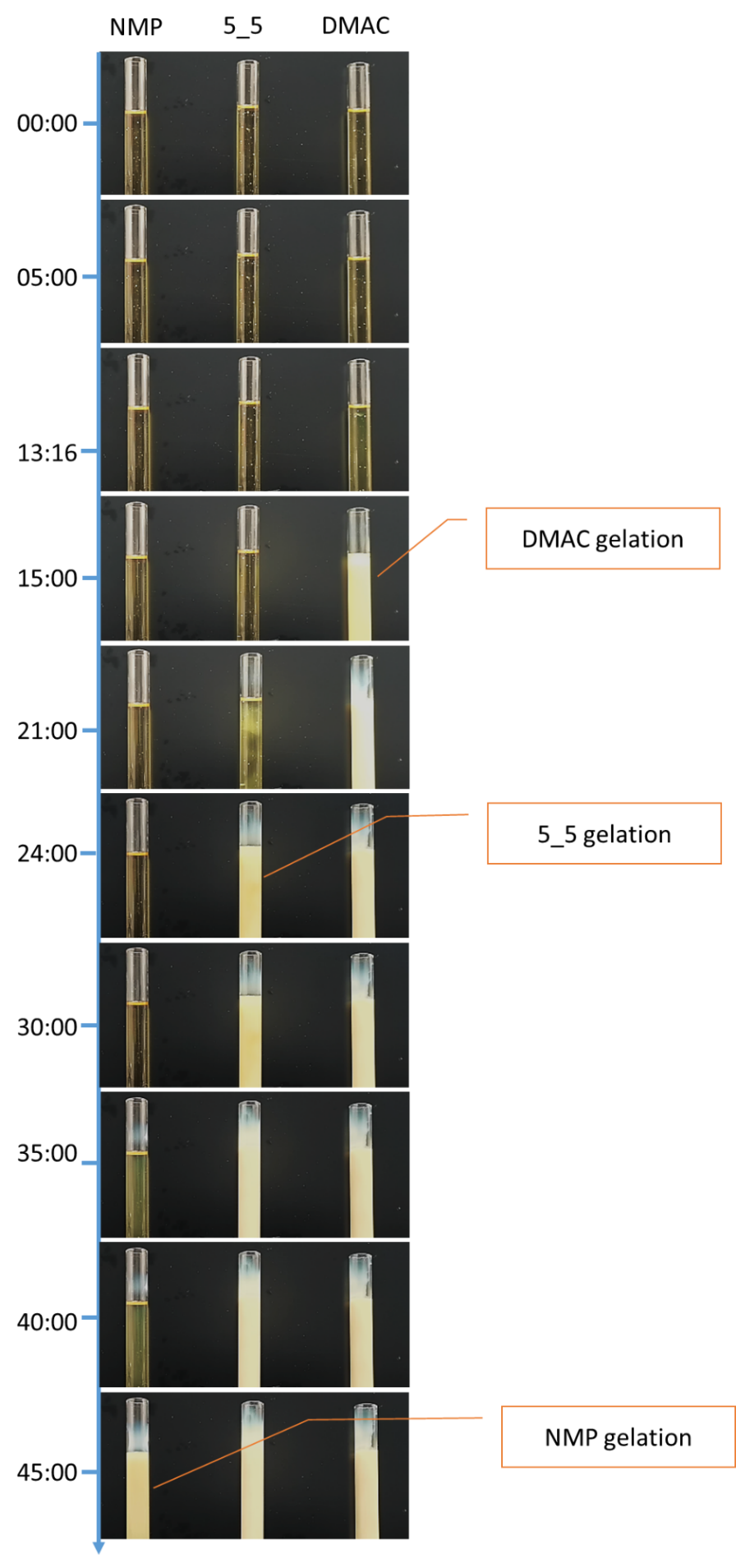


**Figure S6.** Gels formation timelines.


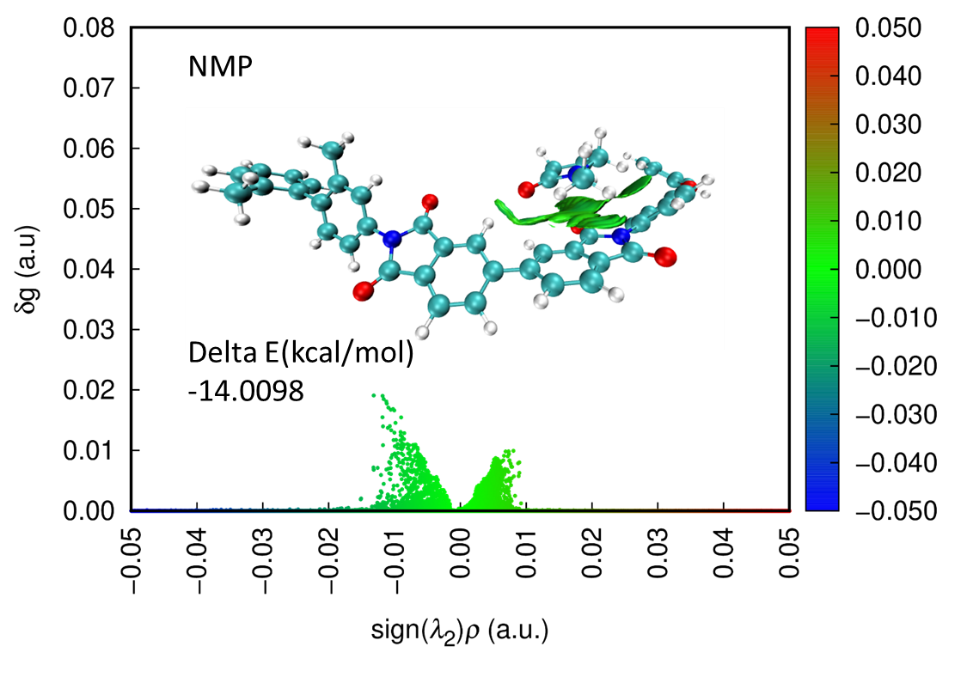


**Figure S7.** Independent Gradient Model based on Hirshfeld partition (IGMH) results of the interaction between defined polyimide structure and NMP.


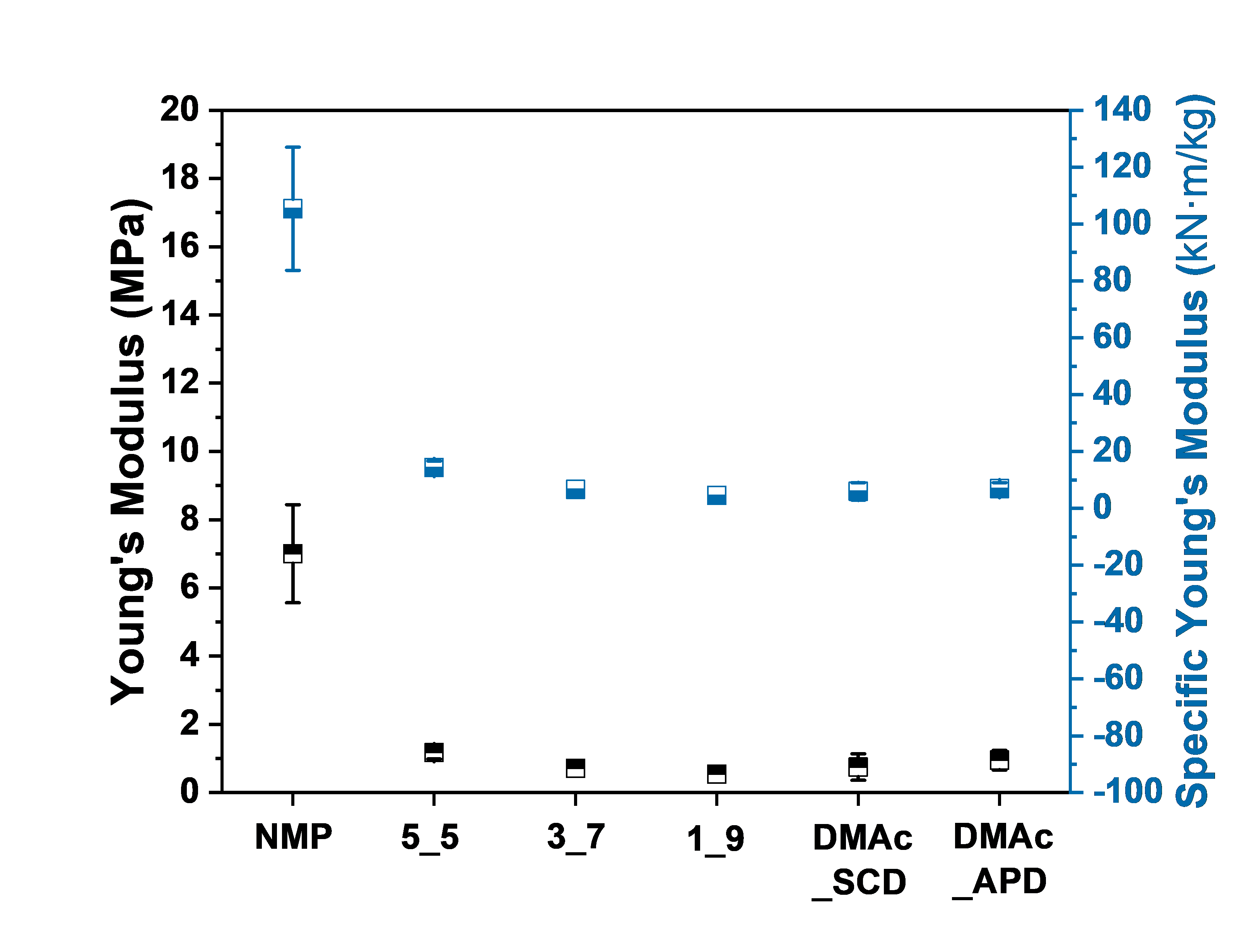


**Figure S8.** Compressive Young’s modulus and specific Young’s modulus.

**
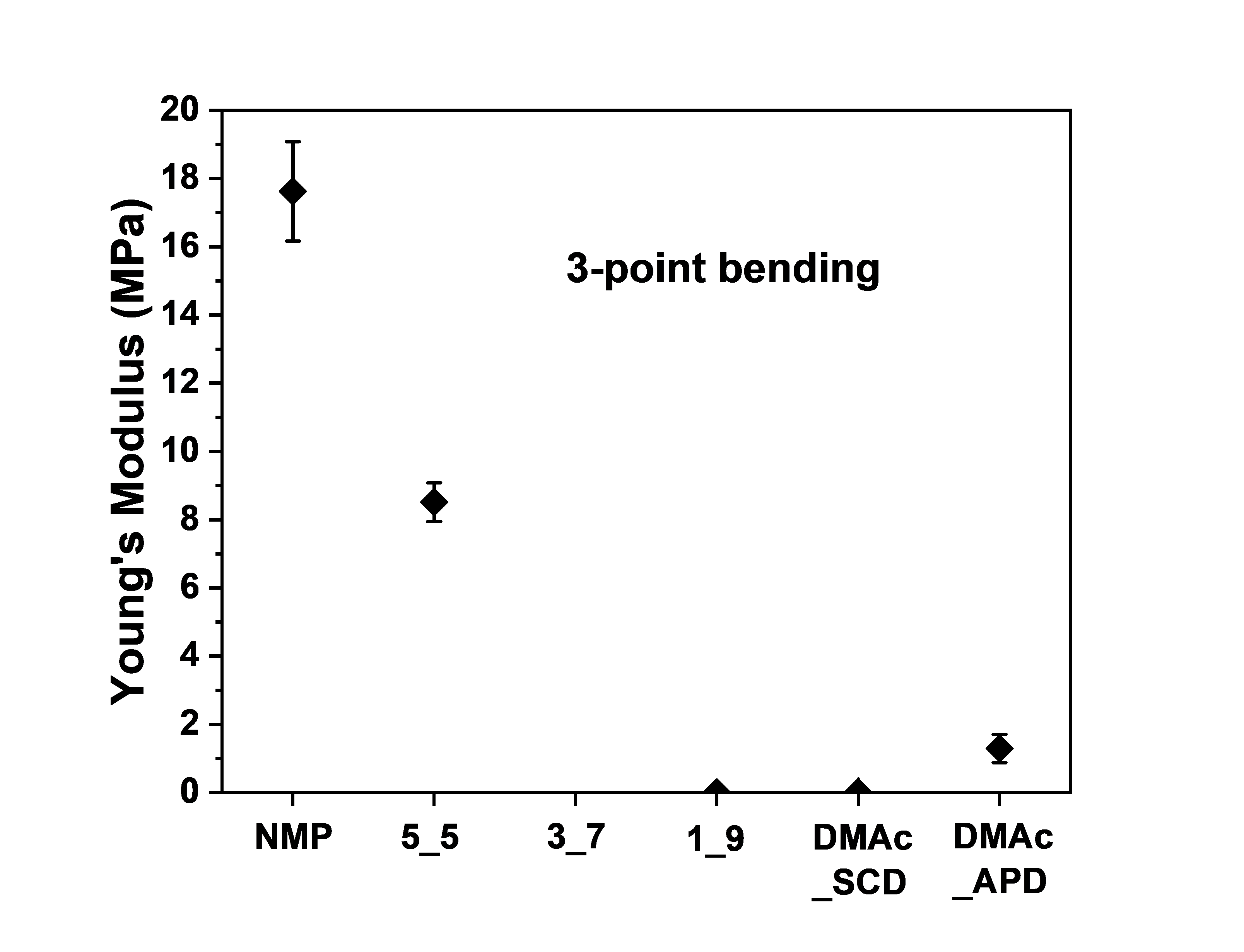
**

**Figure S9.** The Young’s modulus of polyimide aerogels from 3-point bending test.


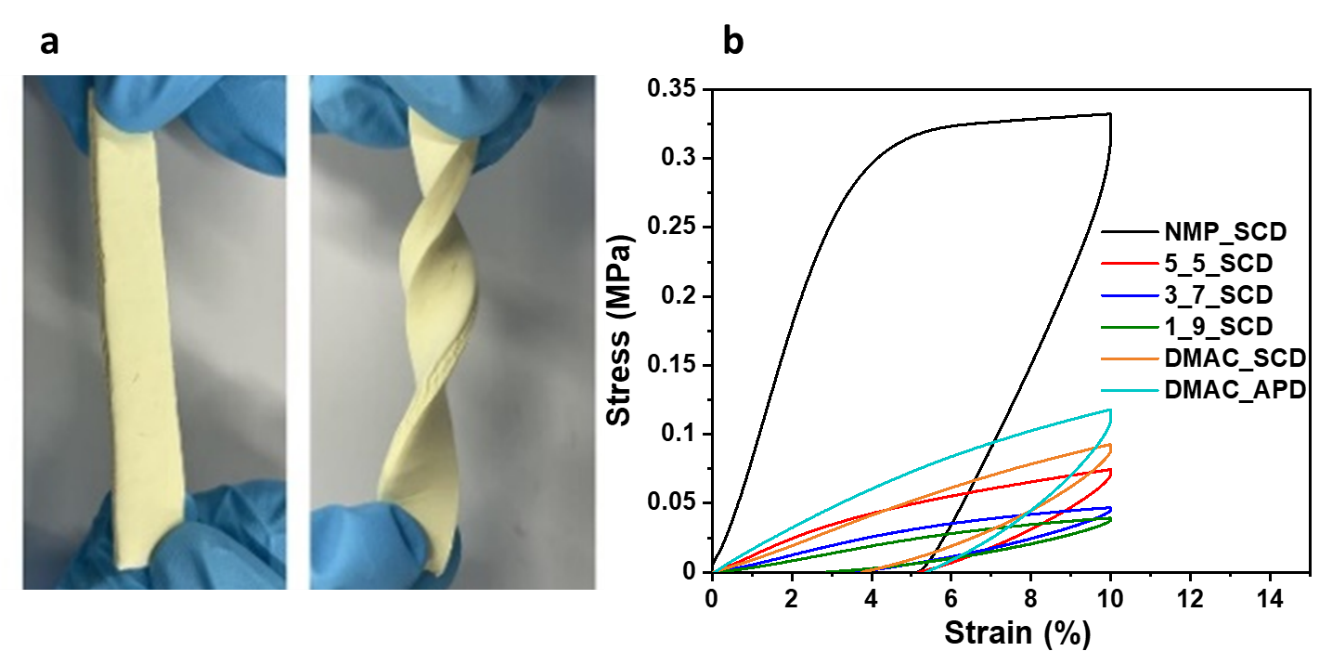


**Figure S10.** Mechanical properties of the polyimide aerogels (a) flexibility of the PI_DMAc_ aerogel, (b) stress-strain curves of aerogels under various conditions.

**
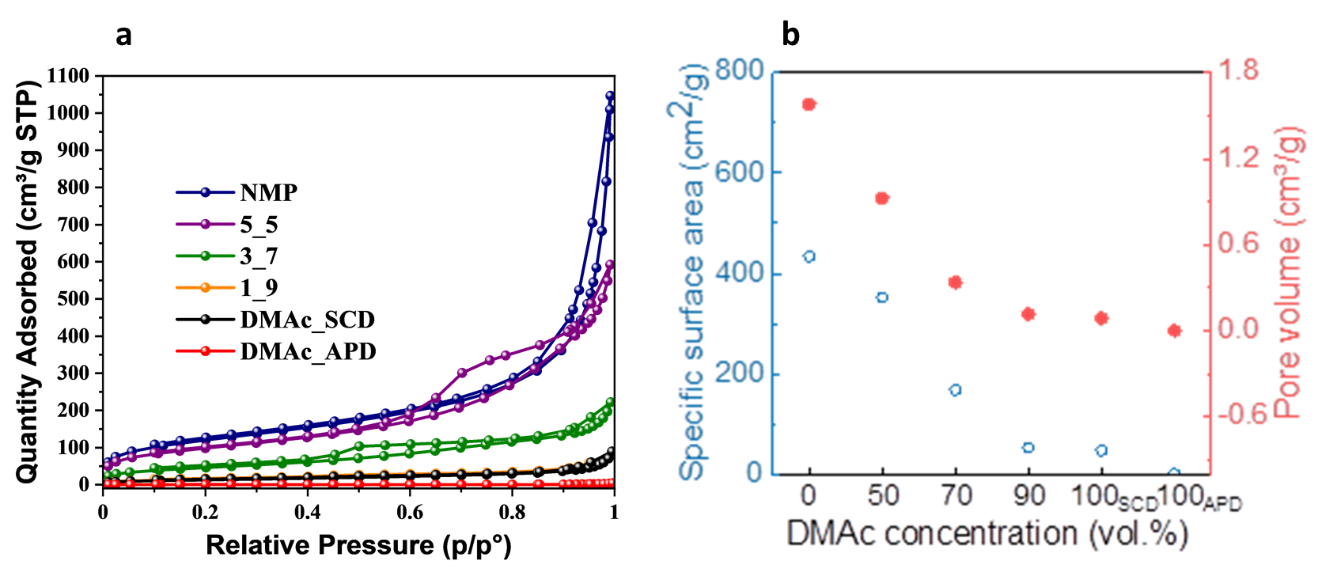
**

**Figure S11.** (a) N_2_ isotherms and (b) specific surface area and pore volume of all the polyimide aerogels under various preparation conditions.

**Figure S12.** The N_2_ isotherms of all the polyimide aerogels dried from different solvents.


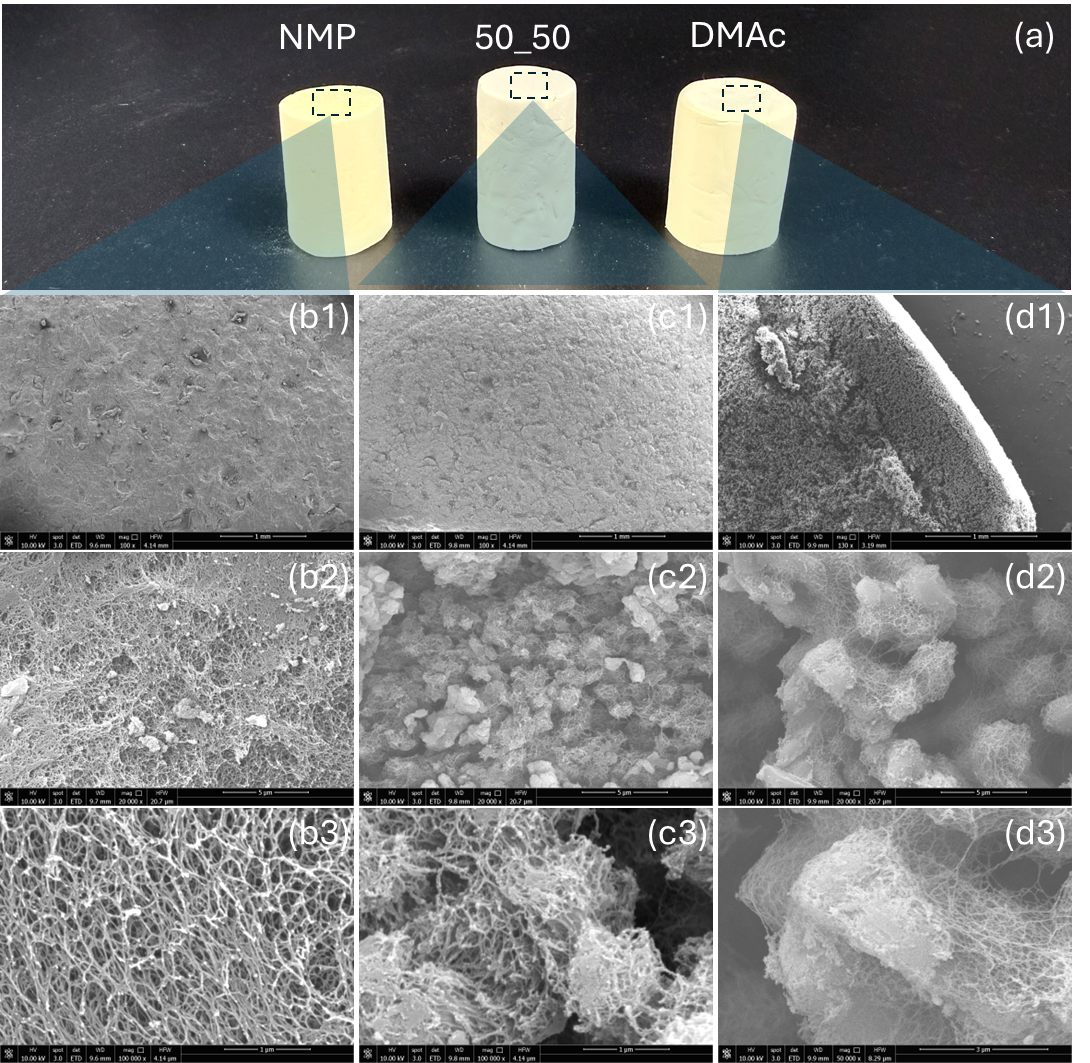


**Figure S13.** Outer surface structures of the samples prepared under different conditions: (a) photographs of samples prepared from NMP, 50NMP_50DMAc, and DMAc solvents, and SEM images at different magnifications of (b1–3) NMP, (c1–3) 50NMP_50DMAc, and (d1–3) DMAc samples.

**
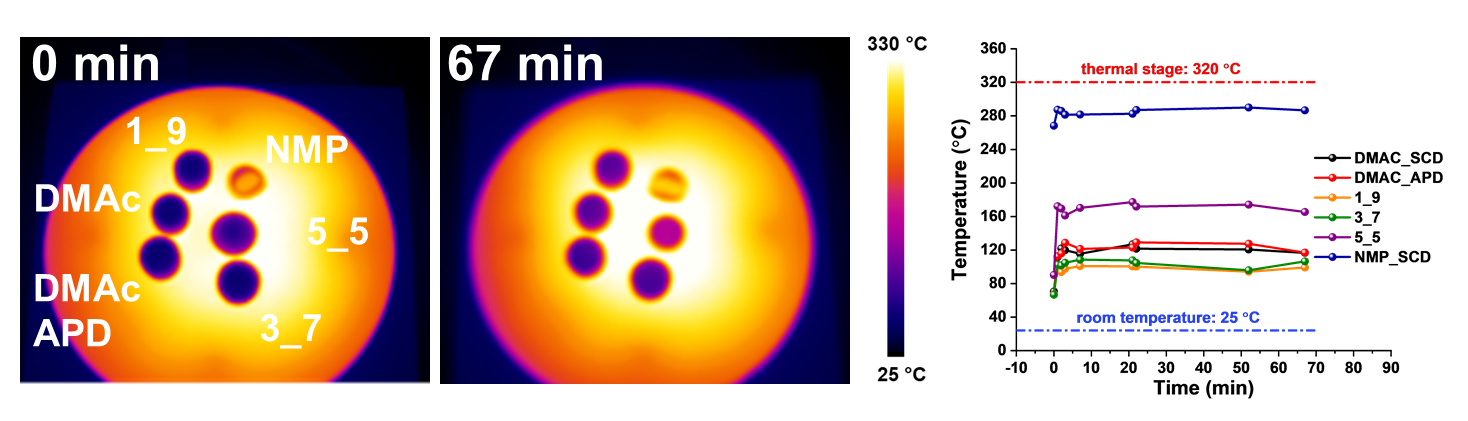
**

**Figure S14.** IR images of six aerogel cylinders (thickness: 10 mm) from different recipes at 0 min and 67 min; temperature evolution of the upper surface of different aerogels with time.

**Table S3.** Comparison of the reported polyimide aerogels: dielectric, thermal conductivity, and density

| **Source** | **Thermal conductivity**  **W/m·K** | **Dielectric properties** | | | **Density (g/cm³)** |
| --- | --- | --- | --- | --- | --- |
|  |  | **Dielectric Constant (ε′)** | **Loss Tangent (tan δ)** | **Frequency Range** |  |
| This study | 0.025-0.035 | 2.0 (<300 kHz)  1.1 (8-12 GHz) | <0.2 | 0.1Hz-300 kHz  8-12GHz | 0.06-0.13 |
| Meador et al. (2012) PI [14] | N/A | 1.16-1.36 | <0.01 | 0.05–40 GHz | 0.10-0.26 |
| Xu et al. (2024) PI [15] | ~0.023 | 1.51–2.42 (100 kHz); 2.48 (10 GHz) | ~0.0025–0.0038 | 100 kHz; 8.2–12.5 GHz | N/A |
| Li et al. (2024) PI [16] | 0.0319 | 1.60 | 0.0171 | 10 MHz | 0.12-0.20 |
| Li et al. (2024) PI [17] | 0.0354 | 1.33 (1 kHz); 1.32 (1 MHz) | 0.0079 (1 kHz); 0.0035 (1 MHz) | 1 kHz; 1 MHz | 0.13–0.21 |
| Li et al. (2022) – Single-crosslinked PI [18] | 0.0599 | 1.34–1.53 (12 GHz) | 0.008–0.020 (12 GHz) | 8–12 GHz | 0.156–0.220 |
| Li et al. (2022) – Double-crosslinked PI [18] | 0.0287 | 1.10–1.17 (12 GHz) | 0.003–0.006 (12 GHz) | 8–12 GHz | 0.085–0.110 |
| Kantor et al. (2022) PI–silica [19] | 0.0175 | 2.5 (10⁻¹–10⁶ Hz); 1.2 (8–12.5 GHz); 1.2 (26.5–32 GHz) | 10⁻³ to 10⁻¹ (all studied frequencies) | 0.1 Hz–32 GHz | 0.112 |
| Wu et al. (2023) PI–silica [20] | 0.0203 | 2.17 (0.1–10⁶ Hz); 1.00–1.50 (X); 1.15–1.17 (Ku–K); 1.20–1.72 (Ka) | 1e-4; ±0.2 (X); –0.03–0.02 (Ku–K); –0.15–0.30 (Ka) | 0.1 Hz–40 GHz | 0.09–0.11 |
| AeroZero®, PI [21] | 0.02–0.04 | <1.40 | 0.0007–0.0030 | 1–50 GHz | ~0.25 |
| Cashman et al. (2020), PI [22] | 0.02–0.03 | 1.11–1.16 | Low (n.d.) | 10 GHz | 0.10–0.30 |
| Meador et al. (2014), PI [23] | N/A | ~1.08 | Low (n.d.) | 11–12 GHz | 0.09–0.20 |
| Tafreshi (2022) PI [24] | 0.0275 | ~2.7 | N/A | 10^−1^–10^5^ Hz | ~0.089 |


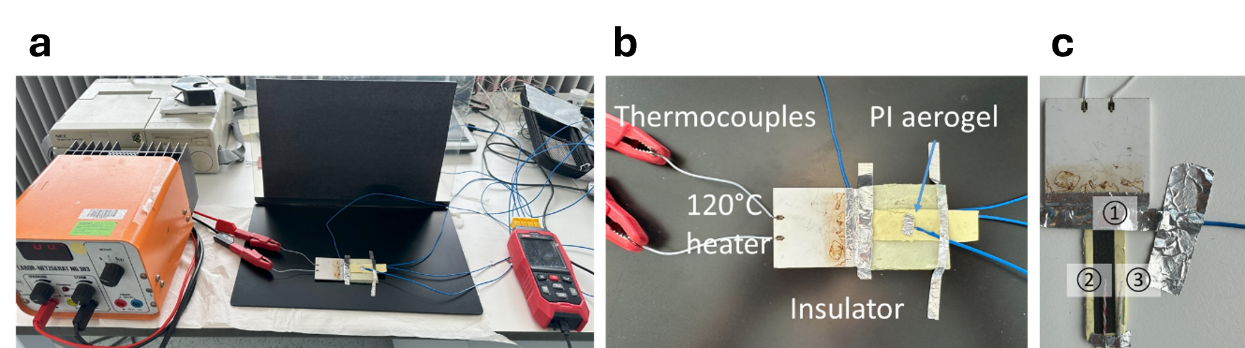


**Figure S15.** The setup for thermal management of LIG-polyimide aerogels.

**Table S4** Parameters used for the optimization of the LIG on polyimide aerogels

| Sample | P (%)* | S (%) | DPI | Defocus (mm) | Dithering | Pass | Resistivity |
| --- | --- | --- | --- | --- | --- | --- | --- |
| 1 | 7 | 5 | 1000 | -5 | fixed | 1 | - |
| 2 | 8 | 4.5 | 1000 | -5 | fixed | 1 | 30 |
| 3 | 7.5 | 4.8 | 1000 | -5 | fixed | 1 | - |
| 4 | 7 | 5 | 500 | -5 | fixed | 2 | - |
| 5 | 7 | 5 | 600 | -5 | fixed | 2 | - |
| 6 | 7.5 | 5 | 600 | -5 | fixed | 2 | - |
| 7 | 7.5 | 4.7 | 500 | -5 | fixed | 2 | - |
| 8 | 7.5 | 4.5 | 600 | -5 | fixed | 2 | - |
| 9 | 7.5 | 4.5 | 600 | -5 | fixed | 3 | - |
| 10 | 8 | 4 | 600 | -5 | fixed | 2 | 6.5 |
| 11 | 8.5 | 4 | 500 | -5 | fixed | 2 | 9 |
| 12 | 8.5 | 4 | 600 | -5 | fixed | 2 | 8.5 |
| 13 | 8 | 6.5 | 1000 | -5 | fixed | 2 | 21 |
| 14 | 8 | 4 | 333 | -5 | fixed | 3 | 15 |
| 15 | 8.5 | 4 | 333 | -5 | fixed | 3 | 10 |
| 16 | 8 | 3.5 | 333 | -5 | fixed | 3 | 9 |
| 17 | 8 | 2.8 | 333 | -5 | fixed | 2 | 35 |
| 18 | 8 | 2.5 | 333 | -5 | fixed | 2 | - |

* P%: laser power (% of maximum output 50W), S%: scanning speed (% of maximum scanning speed, 3000 mm s^-1^), DPI: Dots Per Inch (laser pulse density / resolution), Defocus: Focal offset distance mm, Dithering: Laser modulation pattern/grayscale, Pass: Number of laser scans over the same area.


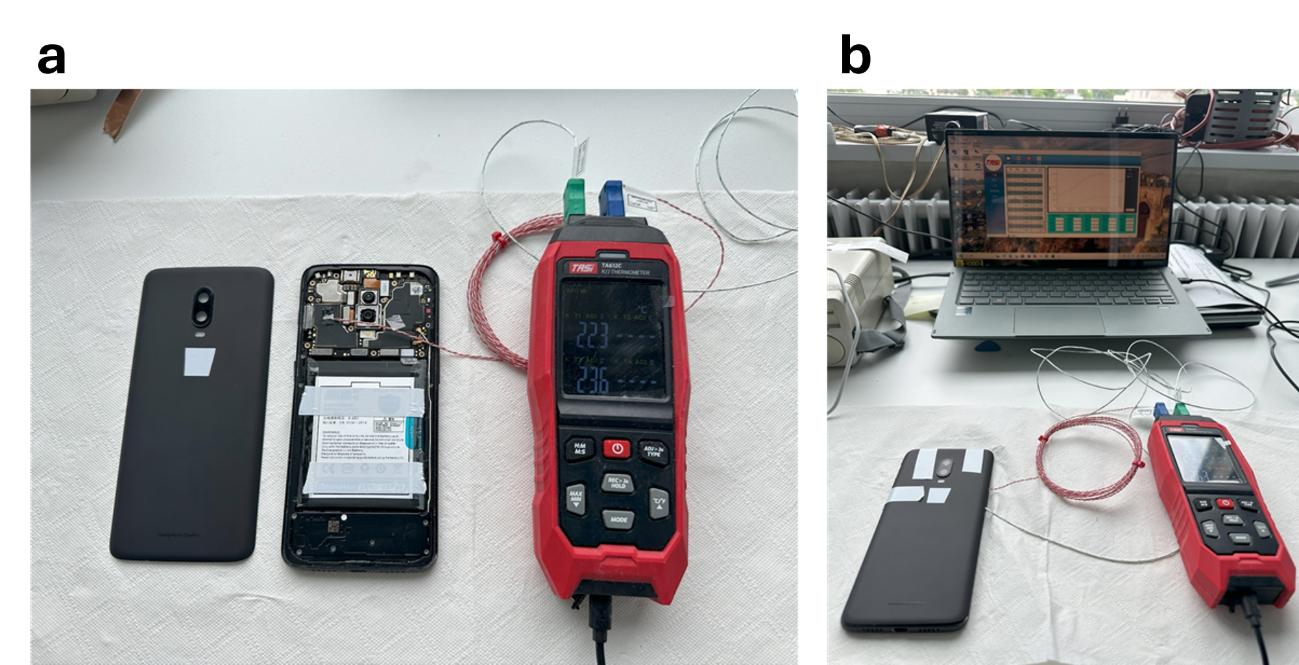


**Figure S16.** The setup for thermal management of LIG-polyimide aerogels on a 5G smartphone.


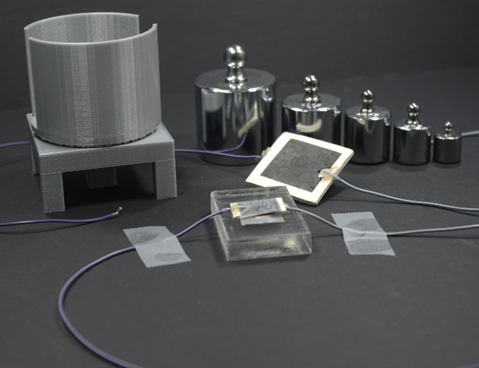

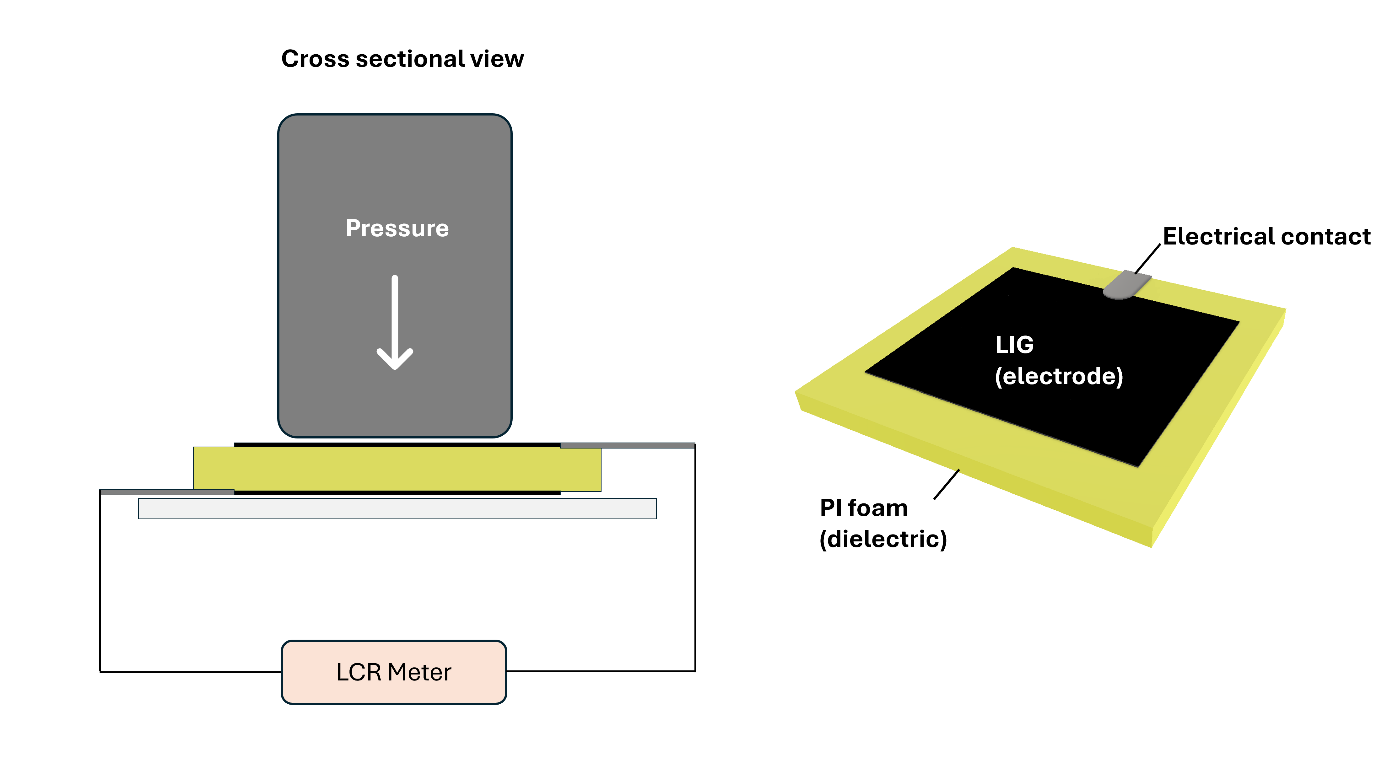


**a**

**b**

**Figure S17.** The setup (a) and schematic representation (b) of LIG-polyimide aerogels as a pressure sensor.

**Table S5**. Calculated value of mass in kg and pressure in mN/mm^2^ for pressure sensor demonstration.

| **Mass in kg** | **Pressure in mN/mm^2^** |
| --- | --- |
| 1 | 43.60 |
| 0.5 | 21.80 |
| 0.2 | 8.72 |
| 0.1 | 4.36 |
| 0.05 | 2.18 |
| 0.02 | 0.87 |


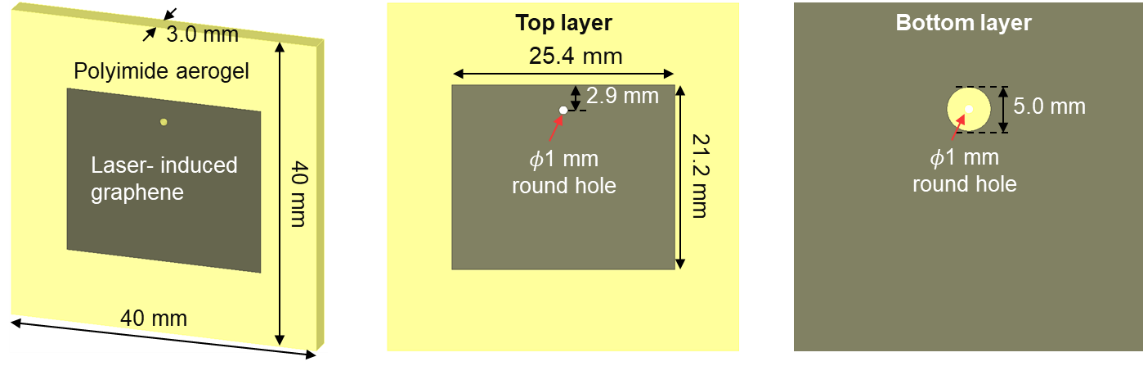


**Figure S18.** Proposed patch antenna design.

**
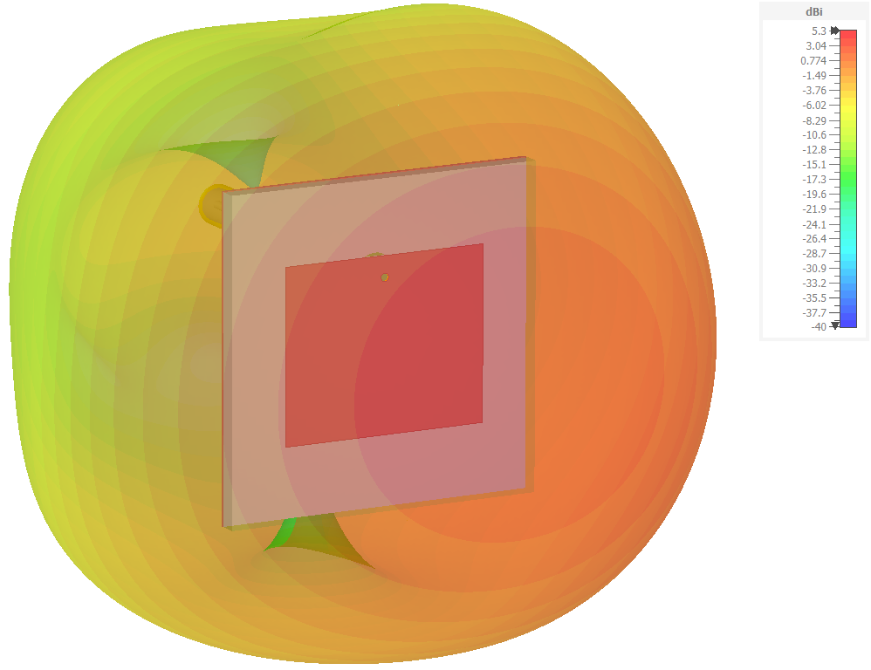
**

**Figure S19.** Simulated 3D radiation pattern of the patch antenna.

Movie S1.

Gels formation with different NMP to DMAc ratios.

Movie S2.

The flexibility of a PI_DMAc_ aerogel with rolling and unrolling.

Movie S3.

Fabrication of a large (20 × 15 cm^2^) PI_DMAc_ blanket by laser writing and cutting.

**Supplementary References**

1. M. J. Frisch, G. W. T., H. B. Schlegel, G. E. Scuseria, M. A. Robb, J. R. Cheeseman, G. Scalmani, V. Barone, G. A. Petersson, H. Nakatsuji, X. Li, M. Caricato, A. V. Marenich, J. Bloino, B. G. Janesko, R. Gomperts, B. Mennucci, H. P. Hratchian, J. V. Ortiz, A. F. Izmaylov, J. L. Sonnenberg, D. Williams-Young, F. Ding, F. Lipparini, F. Egidi, J. Goings, B. Peng, A. Petrone, T. Henderson, D. Ranasinghe, V. G. Zakrzewski, J. Gao, N. Rega, G. Zheng, W. Liang, M. Hada, M. Ehara, K. Toyota, R. Fukuda, J. Hasegawa, M. Ishida, T. Nakajima, Y. Honda, O. Kitao, H. Nakai, T. Vreven, K. Throssell, J. A. Montgomery, Jr., J. E. Peralta, F. Ogliaro, M. J. Bearpark, J. J. Heyd, E. N. Brothers, K. N. Kudin, V. N. Staroverov, T. A. Keith, R. Kobayashi, J. Normand, K. Raghavachari, A. P. Rendell, J. C. Burant, S. S. Iyengar, J. Tomasi, M. Cossi, J. M. Millam, M. Klene, C. Adamo, R. Cammi, J. W. Ochterski, R. L. Martin, K. Morokuma, O. Farkas, J. B. Foresman, and D. J. Fox, Gaussian16, Revision C.01. *Gaussian, Inc., Wallingford CT* **2019**.

2. Lu, T.; Chen, F., Multiwfn: a multifunctional wavefunction analyzer. *Journal of Computational Chemistry* **2012,** *33* (5), 580-92. DOI 10.1002/jcc.22885.

3. Lu, T.; Chen, Q., Independent gradient model based on Hirshfeld partition: A new method for visual study of interactions in chemical systems. *Journal of Computational Chemistry* **2022,** *43* (8), 539-555. DOI 10.1002/jcc.26812.

4. Breßler, I.; Kohlbrecher, J.; Thünemann, A. F., SASfit: a tool for small-angle scattering data analysis using a library of analytical expressions. *J Appl Crystallogr* **2015,** *48* (5), 1587-1598.

5. Zhao, S.; Malfait, W. J.; Demilecamps, A.; Zhang, Y.; Brunner, S.; Huber, L.; Tingaut, P.; Rigacci, A.; Budtova, T.; Koebel, M. M., Strong, Thermally Superinsulating Biopolymer-Silica Aerogel Hybrids by Cogelation of Silicic Acid with Pectin. *Angewandte Chemie International Edition* **2015,** *54* (48), 14282-6. DOI 10.1002/anie.201507328.

6. Aeby, X.; Bourely, J.; Poulin, A.; Siqueira, G.; Nyström, G.; Briand, D., Printed humidity sensors from renewable and biodegradable materials. *Advanced Materials Technologies* **2023,** *8* (5), 2201302.

7. Xie, F.; Li, Z.; Li, Z.; Li, D.; Gao, Y.; Wang, B., Absolute intensity calibration and application at BSRF SAXS station. *Nuclear Instruments and Methods in Physics Research Section A: Accelerators, Spectrometers, Detectors and Associated Equipment* **2018,** *900*, 64-68. DOI <https://doi.org/10.1016/j.nima.2018.05.026>.

8. Zimm, B. H., The Scattering of Light and the Radial Distribution Function of High Polymer Solutions. *The Journal of Chemical Physics* **1948,** *16* (12), 1093-1099. DOI 10.1063/1.1746738.

9. Koberstein, J. T., Polymers and Neutron Scattering. Oxford Series on Neutron Scattering in Condensed Matter No. 8 By Julia S. Higgins (Imperial College, London, U.K.) and Henri C. Benoit (Universite Louis Pasteur, Strasbourg, France). Oxford:  New York. 1994. xix + 436 pp. $98.00. ISBN 0-19-851003-9. *Journal of the American Chemical Society* **1996,** *118* (45), 11338-11339. DOI 10.1021/ja9553415.

10. Takeshita, S.; Sadeghpour, A.; Malfait, W. J.; Konishi, A.; Otake, K.; Yoda, S., Formation of Nanofibrous Structure in Biopolymer Aerogel during Supercritical CO2 Processing: The Case of Chitosan Aerogel. *Biomacromolecules* **2019,** *20* (5), 2051-2057. DOI 10.1021/acs.biomac.9b00246.

11. Chartier, C.; Buwalda, S.; Van Den Berghe, H.; Nottelet, B.; Budtova, T., Tuning the properties of porous chitosan: Aerogels and cryogels. *International Journal of Biological Macromolecules* **2022,** *202*, 215-223. DOI <https://doi.org/10.1016/j.ijbiomac.2022.01.042>.

12. Schubert, K. V.; Strey, R.; Kline, S. R.; Kaler, E. W., Small angle neutron scattering near Lifshitz lines: Transition from weakly structured mixtures to microemulsions. *The Journal of Chemical Physics* **1994,** *101* (6), 5343-5355. DOI 10.1063/1.467387.

13. Teubner, M.; Strey, R., Origin of the scattering peak in microemulsions. *The Journal of Chemical Physics* **1987,** *87* (5), 3195-3200. DOI 10.1063/1.453006.

14. Meador, M. A. B.; Wright, S.; Sandberg, A.; Nguyen, B. N.; Van Keuls, F. W.; Mueller, C. H.; Rodríguez-Solís, R.; Miranda, F. A., Low dielectric polyimide aerogels as substrates for lightweight patch antennas. *ACS Appl. Mater. Interfaces* **2012,** *4* (11), 6346-6353.

15. Li, H.; Kong, X.; Wang, S.; Gong, M.; Lin, X.; Zhang, L.; Wang, D., Sustainable Dielectric Films with Ultralow Permittivity from Soluble Fluorinated Polyimide. *Molecules* **2023,** *28* (7), 3095.

16. Li, D.; Ye, Q.; Zhao, D.; Xie, M.; Li, D.; Yu, Y.; Qian, G.; Chen, C., Construction of polyimide composite aerogels via multi-coordination structure of polymer-metal for improving heat-induced shrinkage, thermal insulation, mechanical and dielectric properties. *Materials Today Communications* **2024,** *39*, 109035.

17. Li, D.; Lu, Z.; Ke, Z.; Xu, K.; Dai, F.; Yu, Y.; Qian, G.; Chen, C., Moisture resistant polyimide aerogel membranes with low dielectric constant and super thermal insulation for electronic device under harsh environment. *Polymer* **2024,** *290*, 126478.

18. Li, Y.; Ma, B.; Zhang, R.; Luo, X., Mechanically strong, thermal-insulated, and ultralow dielectric polyimide aerogels with adjustable crosslinking methods. *Polymer* **2022,** *253*, 125035.

19. Kantor, Z.; Wu, T.; Zeng, Z.; Gaan, S.; Lehner, S.; Jovic, M.; Bonnin, A.; Pan, Z.; Mazrouei-Sebdani, Z.; Zhao, S., Heterogeneous silica-polyimide aerogel-in-aerogel nanocomposites. *Chem Eng J* **2022,** *443*, 136401.

20. Zhao, T. W. M. G. G. S. Z. Z. W. J. M. S., 3D Printed Polyimide Nanocomposite Aerogels for Electromagnetic Interference Shielding and Thermal Management. *Advanced Materials Technologies* **2023,** *8* (14), 202202155. *doi: 10.1002/admt.202202155*.

21. Blueshift Blueshift Materials’ AeroZero® Targets the Surging High-Frequency High-Speed Materials Market, <https://www.blueshiftmaterials.com/blueshift-materials-aerozero-targets-the-surging-high-frequency-high-speed-materials-market/?utm_source=chatgpt.com>. <https://www.blueshiftmaterials.com/blueshift-materials-aerozero-targets-the-surging-high-frequency-high-speed-materials-market/?utm_source=chatgpt.com>.

22. Cashman, J. L.; Nguyen, B. N.; Dosa, B.; Meador, M. A. B., Flexible polyimide aerogels derived from the use of a neopentyl spacer in the backbone. *ACS Applied Polymer Materials* **2020,** *2* (6), 2179-2189.

23. Meador, M. A. B.; McMillon, E.; Sandberg, A.; Barrios, E.; Wilmoth, N. G.; Mueller, C. H.; Miranda, F. A., Dielectric and Other Properties of Polyimide Aerogels Containing Fluorinated Blocks. *ACS Appl. Mater. Interfaces* **2014,** *6* (9), 6062-6068. DOI 10.1021/am405106h.

24. Tafreshi, O. A.; Ghaffari-Mosanenzadeh, S.; Karamikamkar, S.; Saadatnia, Z.; Kiddell, S.; Park, C. B.; Naguib, H. E., Novel, flexible, and transparent thin film polyimide aerogels with enhanced thermal insulation and high service temperature. *Journal of Materials Chemistry C* **2022,** *10* (13), 5088-5108.
